# Biosynthesis of UDP-β-l-Arabinofuranoside for the Capsular Polysaccharides of *Campylobacter jejuni*

**DOI:** 10.1021/acs.biochem.3c00298

**Published:** 2023-09-22

**Authors:** Max Errickson Simons, Tamari Narindoshvili, Frank M. Raushel

**Affiliations:** † Department of Biochemistry & Biophysics, Texas A&M University, College Station, Texas 77842, United States; ‡ Department of Chemistry, Texas A&M University, College Station, Texas 77842, United States

## Abstract

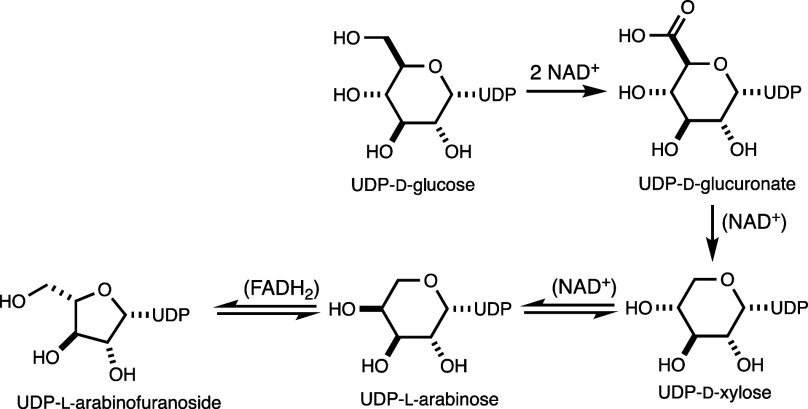

*Campylobacter jejuni* is the leading cause of food poisoning in North America and Europe. The exterior surface of this bacterium is coated with a capsular polysaccharide (CPS) which enables adherence to the host epithelial cells and evasion of the host immune system. Many strains of *C. jejuni* can be differentiated from one another by changes in the sequence of the carbohydrates found within the CPS. The CPS structures of serotypes HS:15 and HS:41 of *C. jejuni* were chemically characterized and found to contain an l-arabinofuranoside moiety in the repeating CPS sequence. Sequence similarity and genome neighborhood networks were used to identify the putative gene cluster within the HS:15 serotype for the biosynthesis of the l-arabinofuranoside fragment. The first enzyme (HS:15.18) in the pathway was found to catalyze the NAD^+^-dependent oxidation of UDP-α-d-glucose to UDP-α-d-glucuronate, while the second enzyme (HS:15.19) catalyzes the NAD^+^-dependent decarboxylation of this product to form UDP-α-d-xylose. The UDP-α-d-xylose is then epimerized at C4 by the third enzyme (HS:15.17) to produce UDP-β-l-arabinopyranoside. In the last step, HS:15.16 catalyzes the FADH_2_-dependent conversion of UDP-β-l-arabinopyranoside into UDP-β-l-arabinofuranoside. The UDP-β-l-arabinopyranoside mutase catalyzed reaction was further interrogated by measurement of a positional isotope exchange reaction within [^18^O]-UDP-β-l-arabinopyranoside.

## Introduction

The most common form of food poisoning in the United States and Europe is caused by an infection with *Campylobacter jejuni*.^1,2^ This Gram-negative bacterium is considered commensal in chickens and campylobacteriosis results from the ingestion of undercooked and contaminated chicken products.^1^ Common symptoms of this infection include diarrhea, fever, and vomiting.^2,3^ The toxic effects induced by *C. jejuni* are thought to be caused by the generation and secretion of a cytolethal distending toxin.^4,5^ This toxin is composed of a heterotrimeric complex of CdtA, CdtB, and CdtC.^4,5^ The cytolethal distending toxin complex has DNase activity resulting in double strand DNA cleavage and cell-cycle arrest of infected cells.^4–6^ In addition to these problems, approximately 1 in every 1000 *C. jejuni* infections give rise to Guillain-Barré Syndrome (GBS), an autoimmune disease associated with muscle weakness and paralysis.^2,7^ Unfortunately, there are no FDA-approved vaccines for *C. jejuni*.

**Figure 1:**
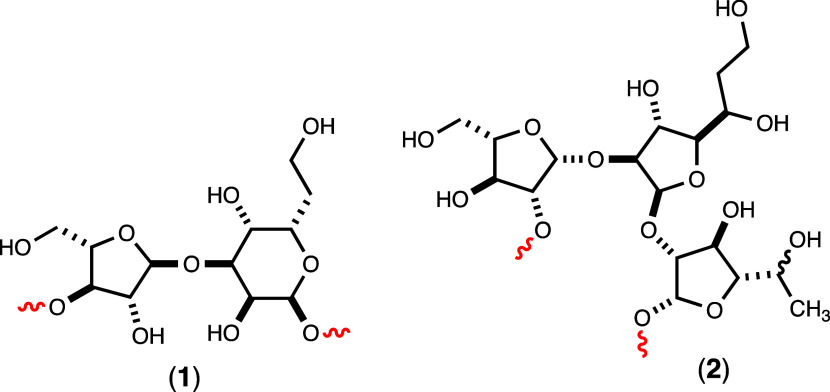
Structures of the repeating polysaccharides in the CPS from serotypes HS:15 (**1**) and HS:41 (**2**) from *C. jejuni*. The repeating polysaccharide in the HS:15 serotype consists of l-arabinose and 6-deoxy-L-*gulo*-heptose. In the HS:41 serotype, the repeating polysaccharide is composed of l-arabinose, 6-deoxy-D-*altro*-heptose, and either d-fucose or 6-deoxy-l-altrose (**2**).

The exterior surface of *C. jejuni* is coated with a capsular polysaccharide (CPS), which is composed of a repeating series of 2–4 different carbohydrates attached to the outer membrane surface through a poly-Kdo (3-deoxy-d-*manno*-oct-2-ulosonic acid) linker.^8^ The individual monosaccharides identified in the CPS are highly variable among different strains and serotypes of *C. jejuni*, allowing for efficient evasion of the host immune system.^9^ To date at least 12 different CPS structures from more than 33 unique serotypes have been chemically characterized.^9–11^ The repeating polysaccharides in the CPS from serotypes HS:15 and HS:41 are highlighted in Figure 1.^12,13^ In the HS:15 serotype the CPS is composed of an alternating sequence of l-arabinose and 6-deoxy-l-*gulo*-heptose.^13^ In contrast, the CPS from the HS:41 serotype is formed from l-arabinose, 6-deoxy-d-*altro*-heptose, and a third sugar, which can either be d-fucose or l-altrose.^12^ We have previously determined the pathway for the biosynthesis of GDP-6-deoxy-β-l-*gulo*-heptose and GDP-6-deoxy-α-d-*altro*-heptose.^10,11^ However, the biosynthesis of the activated forms of l-arabinose, d-fucose, and l-altrose in the HS:15 and HS-41 serotypes is currently unknown.

**Figure 2:**
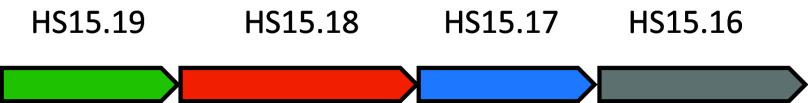
Portion of the gene cluster proposed for the biosynthesis of the activated form of l-arabinofuranoside used in the CPS from the HS:15 serotype from *C. jejuni*. Additional details are provided in the text.

**Figure 3:**
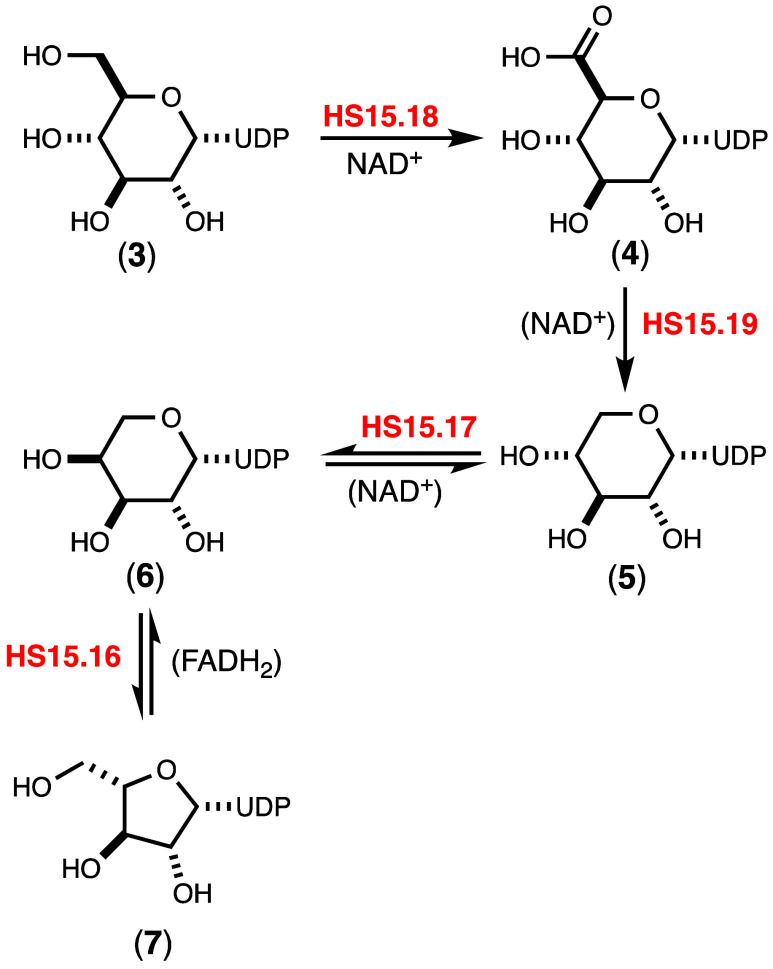
Proposed biosynthetic pathway for the formation of UDP-β-l-arabinofuranoside (**7**) from UDP-α-d-glucose (**3**).

Within the two gene clusters for the biosynthesis of the CPS in the HS:15 and HS:41 serotypes of *C. jejuni* there are four genes that likely contribute to the assembly of the activated form of l-arabinofuranoside (Figure 2). In the HS:15 serotype, these genes include HS15.16 (putative UDP-d-galactopyranose mutase), HS15.17 (putative UDP-d-glucose 4-epimerase), HS15.18 (putative UDP-d-glucose 6-dehydrogenase), and HS15.19 (putative UDP-d-gluconate decarboxylase). From the provisional annotations provided by UniProt, a biosynthetic pathway can be constructed for the transformation of UDP-α-d-glucose to UDP-β-l-arabinofuranoside. The proposed biosynthetic pathway is presented in Figure 3. In the first step, UDP-α-d-glucose (**3**) is oxidized by an NAD^+^-dependent 6-dehydrogenase to form UDP-α-d-glucuronate (**4**). This reaction is followed by the decarboxylation of **4** to generate UDP-α-d-xylose (**5**). A 4-epimerase then catalyzes the racemization of C4 to produce UDP-β-l-arabinose (**6**). In the final step, the mutase catalyzes the FADH_2_-dependent equilibration of UDP-β-l-arabinopyranoside and UDP-β-l-arabinofuranoside. In this paper, we have functionally characterized the four enzymes needed for the enzymatic transformation of UDP-α-d-glucose (**3**) to UDP-β-l-arabinofuranoside (**7**) from the HS:15 serotype of *C. jejuni*.

## Materials and Methods

### Materials

Luria broth and isopropyl β-d-1-thiogalactopyranoside (IPTG) were purchased from Research Products International as well as reagents for Terrific Broth (TB) medium. HEPES, triethanolamine, ammonium acetate, UTP, glucose 6-phosphate, sodium pyruvate, and NAD^+^ were purchased from Sigma-Aldrich, in addition to the enzymes pyrophosphatase, lactate dehydrogenase, and phosphoglucomutase. The 1 and 5 mL HiTrap columns and 5 mL Nickel NTA HisTrap columns and Vivaspin 500s and Vivaspin 20 10 kDa filters were purchased from GE Healthcare. The PA1 Dionex Carbopac guard columns (4 × 50 mm) and Dionex Carbopac PA1 analytical columns (4 × 250 mm) were purchased from Thermo-Scientific. The 0.2 μm 47 mm nylon filters for the PA1 columns were purchased through VWR from the PALL Corporation. Sodium dithionite was purchased from Thermo-Fischer. UDP-d-glucuronate was obtained from Biosynth Carbosynth. Oxygen-18 labeled water (97%) was purchased from Medical Isotopes Inc. UDP-β-l-arabinofuranoside (**7**) was chemically synthesized by the modification of previously published procedures as outlined in the Supporting Information.

### Plasmid Construction

The genes for the expression of HS:15.19 (UniProt id: F2X7B3), HS:15.18 (UniProt id: F2X7B2), and HS:15.17 (UniProt id:F2X7B1) were chemically synthesized by Twist Biosciences with an N-terminal hexa-histidine purification tag contained within a pET28 vector. The gene for production of HS:15.16 (UniProt id: F2X7B0) was also synthesized by Twist Bioscience and subsequently subcloned into a pET30 vector with a C-terminal hexa-histidine purification tag. The codons for the first 49 amino acids of HS:15.16 were truncated to correct an apparent misannotation of the proper start codon. The gene for expression of UDP-α-d-glucose pyrophosphorylase from *Leishmania major* (Accession id: EU249268.1) was also synthesized by Twist Bioscience and inserted into a pET29 vector. The protein sequences for all enzymes used in this investigation are listed in Figure S1.

### Protein Expression and Purification

Plasmids containing the genes for the expression of HS:15.16, HS:15.17, HS:15.18, and HS:15.19 and UDP-glucose pyrophosphorylase were transformed into *Escherichia coli* BL21(DE3) competent cells by electroporation. One L of LB and TB cultures were inoculated with 1.0 mL starter cultures of LB, which were originally inoculated from single colonies and grown at 37 °C overnight in the presence of 50 μg/mL kanamycin. The cells were incubated at 37 °C and brought to an OD_600_ of ∼0.6 before being induced with 1.0 mM IPTG. The *E. coli* BL21 cells transformed with plasmids containing HS:15.19 and HS:15.17 were grown at 16 °C, while cells transformed with HS:15.18 and HS:15.16 were grown at 21 °C. The medium for production of HS:15.16 also contained 1.0 mM dithiothreitol. All cells were grown in LB except for HS:15.18, which was grown in a TB medium. After 16 h, the cells were centrifuged at 15,000 rcf for 15 min, followed by resuspension in Buffer A (50 mM HEPES, 20 mM imidazole, 250 mM KCl, pH 8.5), followed by sonication for 1 h with the duty cycle at 55 and the output control at 4.5 on a Branson Sonifier 450 and supplemented with 5 mg of DNase I. HS:15.16 was sonicated with 1.0% TritonX in the lysate. Lysates were centrifuged at 15,000 rcf for 15 min at 4 °C and passed through a Whatman 0.45 μm filter. The supernatant fluid was injected onto a 5 mL GE Healthcare HisTrap nickel affinity column using Buffer A and eluted with a linear gradient of Buffer B (50 mM HEPES, 500 mM imidazole, and 250 mM KCL, pH 8.5). The fractions containing the enzyme were pooled and then dialyzed with GE Healthcare Vivaspin 20 10 kDa filters using 50 mM HEPES, pH 8.5. The enzymes HS:15.16, HS:15.17, HS:15.18, and HS:15.19 were concentrated to 1.3, 4.5, 8.2, and 5.2 mg/mL, respectively, before being flash frozen in liquid nitrogen and stored at −80 °C. SDS-PAGE analysis of the purified proteins is presented in Figure S2.

### Determination of Reaction Rates

UDP-d-glucose 6-dehydrogenase (HS:15.18) was assayed in a solution containing 50 mM triethanolamine (pH 8.7) by following the change in absorbance at 340 nm using a Spectrophotomax Plus plate reader at 25 °C. The concentration of UDP-d-glucose was varied from 0.01 to 0.4 mM at a fixed concentration of 1.0 mM NAD^+^ and the concentration of NAD^+^ was varied from 0.01 to 2.0 mM at a fixed concentration of 1.0 mM UDP-d-glucose using 100 nM enzyme. The kinetic data were fit to eq 1 where *v* is the initial velocity, *E*_t_ is the enzyme concentration, *k*_cat_ is the turnover number, *A* is the substrate concentration, and *K*_m_ is the Michaelis constant.
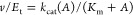
1

UDP-d-glucuronate 6-decarboxylase (HS:15.19) was assayed by following the formation of UDP-d-xylose (**5**) after the decarboxylation of UDP-d-glucuronate (**4**) by using anion exchange chromatography to separate the newly formed product from the remaining substrate. The assays were conducted in a volume of 1.2 mL containing 50 mM phosphate buffer at pH 6.5 using 300 nM enzyme. UDP-d-glucuronate (**4**) was varied from 60 to 3000 μM at a fixed concentration of 0.30 mM NAD^+^. To determine the activation constant (concentration at 1/2 saturation) for NAD^+^, the UDP-d-glucuronate concentration was fixed at 2.5 mM and NAD^+^ was varied from 2.5 to 1500 μM. Aliquots were removed periodically and compounds **4** and **5** were separated from one another using a linear gradient of KCl in 10 mM triethanolamine buffer, pH 8.0, with a 1 mL Hi-Trap anion exchange column (GE Healthcare). Product formation was evaluated using a Bio-Rad NGC chromatography HPLC system with a single wavelength UV–vis detector at 255 nm.

UDP-d-xylose 4-epimerase was assayed by monitoring the formation of UDP-l-arabinose (6) as a function of time. The reactions were conducted in 50 mM HEPES/KOH buffer, pH 8.0, at 25 °C with 5.0 nM enzyme. The separation of UDP-d-xylose (**5**) and UDP-l-arabinose (**6**) was achieved by chromatography on a Carbopac PA1 column using a linear gradient of 60 to 340 mM potassium acetate. The UDP-d-xylose concentration was varied from 14 to 6.0 × 10^3^ μM.

The apparent rate for the reaction catalyzed by UDP-β-l-arabinopyranoside mutase was assayed in the reverse direction starting with the chemically synthesized UDP-β-l-arabinofuranoside (**7**) at a concentration of 1.20 mM in 45 mM HEPES buffer at pH 6.85, 25 °C. The enzyme was utilized at a concentration of 90 nM and 25 μL aliquots were removed as a function of time over a period of 4 min. The reactions were stopped by the addition of 20 μL ethanol^14^ and then lyophilized prior to rehydration and injection onto the column. The UDP-β-l-arabinopyranoside (**6**) was separated from the UDP-β-l-arabinofuranoside (**7**) by chromatography using a column of Carbopac PA1 with a gradient of 0–30% 2 M ammonium acetate.

### Preparation of 18-Oxygen Labeled UDP-α-l-Arabinose (8)

The oxygen-18 labeled UDP-d-xylose was prepared according to the reactions highlighted in Figure S3. Initially, a 1.0 mL solution of 84 mM d-glucose-6-phosphate containing 97% 18-oxygen water was incubated at 55 °C for 48 h to exchange the anomeric oxygen with the 18-oxygen label.^15^ ICP-mass spectrometry demonstrated a labeling of 18-oxygen of 96%. In the next step, 8.0 mM of the 18-oxygen labeled d-glucose-6-phosphate was incubated in a volume of 1.0 mL with 3.0 μM phosphoglucomutase, 4.0 μM UDP-glucose pyrophosphorylase, and 60 nM pyrophosphatase, 2.8 μM lactate dehydrogenase, and 23 μM UDP-d-glucose 6-dehydrogenase (HS:15.18) containing 2.0 mM MgCl_2_, 1.0 mM NAD^+^, 100 mM pyruvate, and 8.0 mM UTP in 50 mM HEPES, pH 8.5, for 17 h. The [^18^O]-UDP-d-glucuronate was purified on a GE Healthcare 5 mL Hi-Trap column with an overall yield of 52% after lyophilization. This material was reconstituted in 0.5 mL of 50 mM HEPES, pH 8.5, and then decarboxylated by the catalytic activity of 20 μM UDP-α-d-glucuronate 6-decarboxylase (HS:15.19) in the presence of 50 μM NAD^+^ to quantitatively form [^18^O]-UDP-d-xylose. The [^18^O]-UDP-d-xylose was converted to an equilibrium mixture with [^18^O]-UDP-l-arabinopyranoside (**8**) by the addition of 5.0 nM UDP-α-d-xylose 4-epimerase (HS:15.17).

The positional isotope exchange (PIX) reactions were conducted at pH 8.5 in 50 mM HEPES buffer at 25 °C. The first reaction was conducted using an equilibrium mixture of the [^18^O]-labeled UDP-d-xylose and UDP-l-arabinose at a total concentration of 4.0 mM in the presence of 20 mM sodium dithionite and 1.25 μM UDP-β-l-arabinopyranoside mutase (HS:15.16). The reaction was allowed to proceed for 3.0 h prior to acquisition of the ^31^P NMR spectrum. In the second experiment, the same conditions were applied to a solution containing 4.0 mM UDP-d-xylose in the absence of UDP-l-arabinose. Control experiments were conducted in the absence of added sodium dithionite to determine whether the tightly bound FAD must be reduced to catalyze the PIX reaction.

### Determination of Tightly Bound Nucleotides

The purified UDP-α-d-xylose 4-epimerase (HS:15.17) was found to have NAD^+^ tightly bound to the active site. The as-purified enzyme was heat-denatured, and after the precipitated protein was removed by centrifugation, the supernatant solution was applied to a 1.0 mL anion exchange column. Nucleotides were eluted from the column using a gradient of 0–2 M KCl in 10 mM triethanolamine buffer at pH 8.0, and the absorbance was monitored at 255 nm. The retention time of the nucleotide isolated from UDP-α-d-xylose 4-epimerase matched that of NAD^+^. The identity of NAD^+^ was confirmed by negative ion ESI-MS with a *m*/*z* for the M-1 anion of 662.10. A similar procedure was used to confirm the binding of FAD to the active site of the as-purified UDP-β-l-arabinopyranoside mutase. The FAD was confirmed by negative ion ESI-MS with an *m*/*z* of 784.15 for the M-1 anion.

## Results and Discussion

### UDP-α-d-Glucose 6-Dehydrogenase

**Figure 4:**
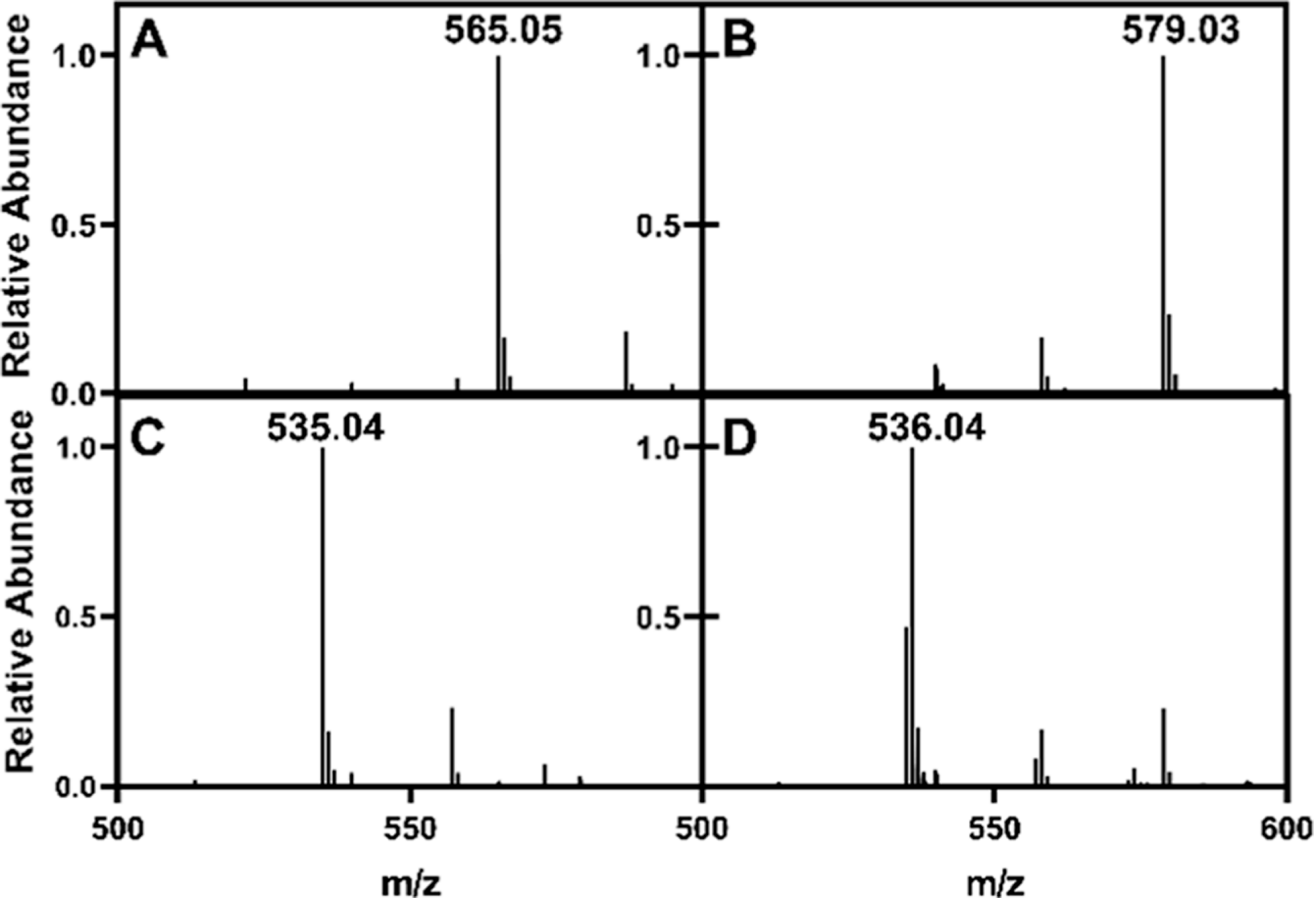
ESI-MS of the products formed by the catalytic activity of UDP-d-glucose 6-dehydrogenase and UDP-d-glucuronate decarboxylase. (A) UDP-d-glucose (**3**) and (B) UDP-d-glucuronate (**4**) formed from the oxidation of **3**. (C) UDP-d-xylose (**5**) formed from the decarboxylation of **4**. (D) [5-^2^H]-UDP-d-xylose (**5**) formed from the enzymatic decarboxylation of **4** in D_2_O.

The first step in the proposed pathway shown in Figure 3 for the formation of UDP-β-l-arabinofuranose (**7**) from UDP-α-d-glucose (**3**) is catalyzed by UDP-α-d-glucose 6-dehydrogenase (HS15.18). The gene for this enzyme from the HS:15 serotype of *C. jejuni* was chemically synthesized and the protein purified to homogeneity using a polyhistidine tag after production in *E. coli*. The purified protein was shown to catalyze the NAD^+^-dependent oxidation of UDP-α-d-glucose (**3**) to UDP-α-d-glucuronate (**4**) and the product was confirmed by ESI-mass spectrometry. The starting material (**3**) has an *m*/*z* of 565.05 for the M-1 anion and the isolated product has an *m*/*z* of 579.03, consistent with the conversion of the –CH_2_OH group to –CO_2_H for a net gain of ∼14 amu (Figure 4a,b). The apparent steady state kinetic constants were determined at pH 8.7 by variation of substrate **3** at a fixed concentration of NAD^+^ and by variation of NAD^+^ at a fixed concentration of **3**. The apparent values of *k*_cat_, *K*_NAD_, *k*_cat_/*K*_NAD_, *K*_UDP-glu_, and *k*_cat_/*K*_UDP-glu_ were determined to be 1.1 ± 0.1 s^–1^, 120 ± 10 μM, 9.1 ± 0.2 × 10^3^ M^–1^ s^–1^, 21 ± 2 μM, and 5.5 ± 0.5 × 10^4^ M^–1^ s^–1^, respectively (Figure S4). The ^1^H NMR spectrum of the product (**4**) is presented in Figure S5.

### UDP-α-d-Glucuronate 6-Decarboxylase

The next step in the proposed biosynthetic pathway for the formation of UDP-β-l-arabinofuranoside (**7**) is the NAD^+^-dependent decarboxylation of **4** to generate UDP-α-d-xylose (**5**). In this transformation substrate **4** is oxidized at C4 by NAD^+^ to generate a UDP-4-keto-α-d-xylose intermediate that is susceptible to decarboxylation.^16^ After decarboxylation, the C5-anion is protonated, followed by the reduction of the 4-keto group by the newly formed NADH (Figure S6). The isolated UDP-α-d-glucuronate 6-decarboxylase (HS15.19) did not contain any tightly bound cofactors, but the enzyme was shown to catalyze the decarboxylation of **4** in the presence of added NAD^+^ to form product **5**. The ESI-mass spectrum of the isolated product (**5**) exhibited an *m*/*z* of 535.05 for the M-1 anion, showing the expected loss of 44 amu for the decarboxylation reaction (Figure 4c) of **4** to **5**. When the reaction was conducted in D_2_O the observed *m*/*z* of the product was 536.04 showing the incorporation of a single deuterium at C5 of compound **5** (Figure 4d). The ^1^H NMR spectra of the isolated product formed in H_2_O and D_2_O are presented in Figure S7. These results support the proposed mechanism in Figure S6. The kinetic constants for the decarboxylation reaction were determined at a fixed concentration of added NAD^+^ (0.3 mM) while varying the concentration of substrate **4**. The apparent values of *k*_cat_, *K*_UDP-glc_, and *k*_cat_/*K*_UDP-glc_ were determined to be 2.6 ± 0.1 s^–1^, 250 ± 30 μM, and 1.1 ± 0.1 × 10^4^ M^–1^ s^–1^, respectively (Figure S8). The activation constant (*K*_a_) for NAD^+^, determined at a fixed concentration of 2.5 mM UDP-α-d-glucuronate (**4**), was determined to be 17 ± 5 μM.

### UDP-α-d-Xylose 4-Epimerase

**Figure 5:**
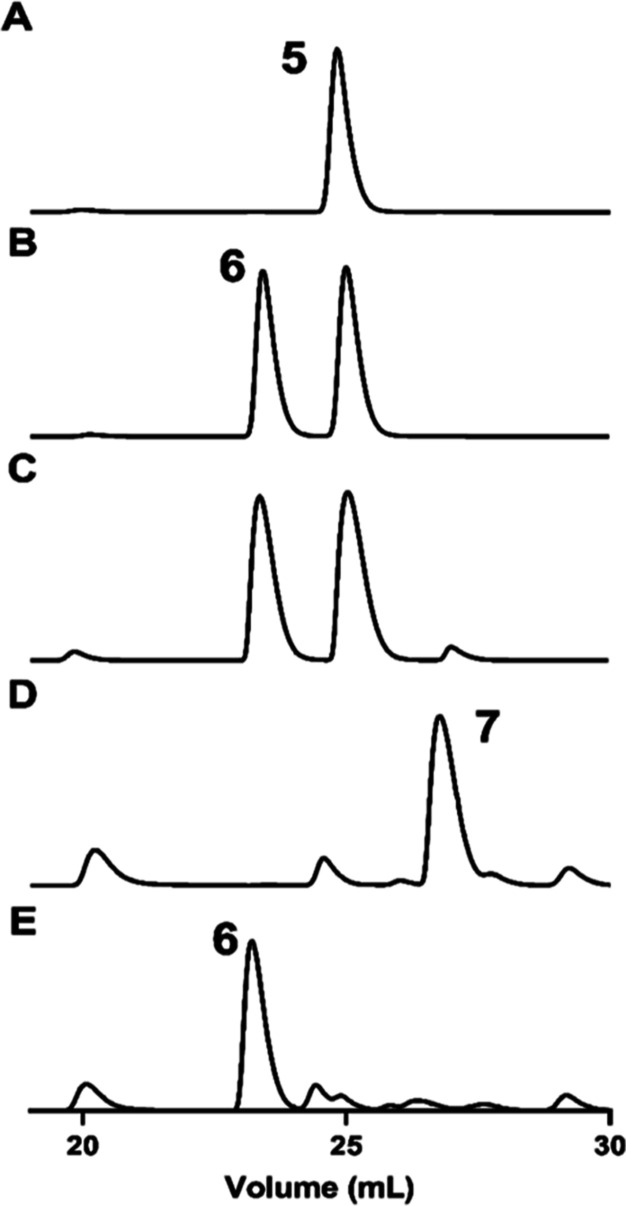
Separation of substrates and enzymatically produced products via chromatography using a CarboPac PA1 HPLC column. The elution profiles were monitored at 255 nm. (A) UDP-d-xylose (**5**) formed from the decarboxylation of **4**. (B) Equilibrium mixture of UDP-d-xylopyranoside (**5**) and UDP-l-arabinopyranoside (**6**). (C) Equilibrium mixture of **5**, **6**, and **7** formed from the addition of UDP-β-l-arabinopyranoside mutase to a mixture of **5** and **6**. (D) Chemically synthesized UDP-β-l-arabinofuranoside (**7**). (E) UDP-β-l-arabinopyranoside (**6**) formed from the addition of UDP-β-l-arabinopyranoside mutase to UDP-β-l-arabinofuranoside (**7**).

The putative 4-epimerase (HS:15.17) required for the interconversion of UDP-α-d-xylose (**5**) and UDP-β-l-arabinose (**6**) was purified to homogeneity and functionally characterized. The isolated enzyme was shown to contain 1 equiv of tightly bound NAD^+^. Incubation of the enzyme with **5** resulted in the formation of **6**. The product and substrate were separated chromatographically using a CarboPac PA1 column (Figure 5a,b). The ^1^H NMR spectrum for the equilibrium mixture of **5** and **6** is presented in Figure S9. The kinetic constants for the catalytic activity of UDP-α-d-xylose 4-epimerase were determined by measuring the rate of formation of **6** by chromatographically separating this product from substrate **5**. The apparent rate constants for the interconversion of **5** and **6** were determined to be *k*_cat_ = 30 ± 6 s^–1^, *K*_UDP-xyl_ = 125 ± 13 μM, and *k*_cat_/*K*_UDP-xyl_ = 2.4 ± 0.2 × 10^5^ M^–1^ s^–1^ (Figure S10).

### UDP-β-l-Arabinopyranoside Mutase

The fourth enzyme in the pathway is expected to interconvert UDP-β-l-arabinopyranoside (**6**) to UDP-β-l-arabinofuranoside (**7**). The enzyme (HS:15.16) was purified to homogeneity and shown to contain ∼1 equiv of tightly bound FAD. The visible spectrum of the isolated enzyme is presented in Figure S11 with an absorbance maximum of ∼450 nm. Previously characterized enzymes that catalyze pyranose to furanose interconversions have been shown to require the flavin to be reduced for maximum catalytic activity.^15,17–19^ The enzyme was reduced with 20 mM sodium dithionite and subsequently shown to catalyze the conversion of **6** into **7** (Figure 5c) by monitoring the formation of **7** after separation of the substrate and product by chromatography with the CarboPac PA1 column. The equilibrium constant favors **6** over **7** by a ratio of approximately 16:1. Since the equilibrium constant for this reaction overwhelmingly favors the pyranose form of the substrate, furanose product **7** was synthesized chemically and used to monitor the reaction in the reverse direction (Figure 5d,e). This experiment clearly demonstrates the formation of **6** from **7**. At an initial concentration of 1.2 mM UDP-β-l-arabinofuranoside (**7**) the apparent value of *k*_cat_ was determined to be 3.9 ± 0.2 s^–1^ at pH 6.8.

### Reaction Mechanism of UDP-β-l-Arabinopyranoside Mutase

**Figure 6:**
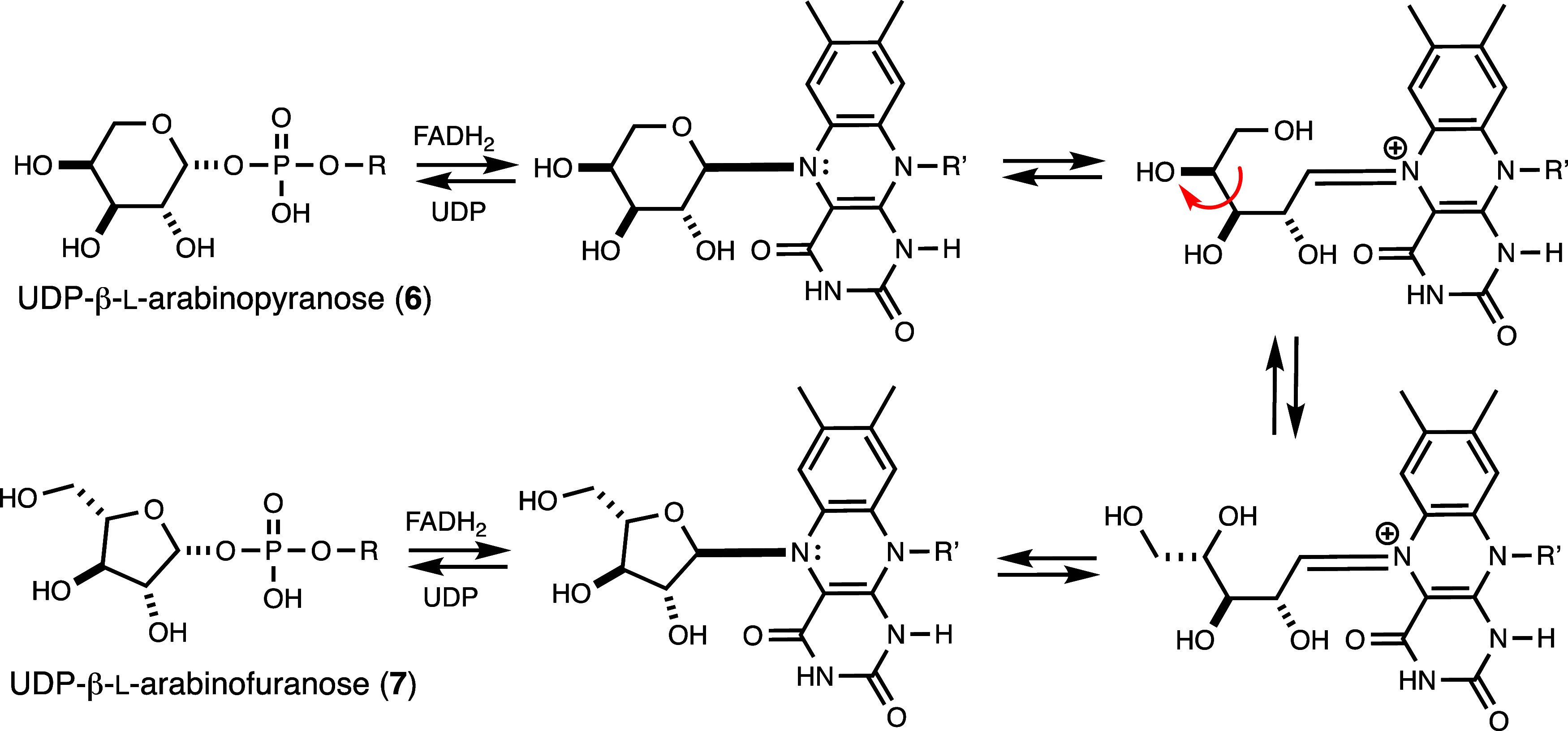
Proposed reaction mechanism for the conversion of **6** into **7** by the UDP-β-l-arabinopyranoside mutase.^15,17–19^

**Figure 7:**
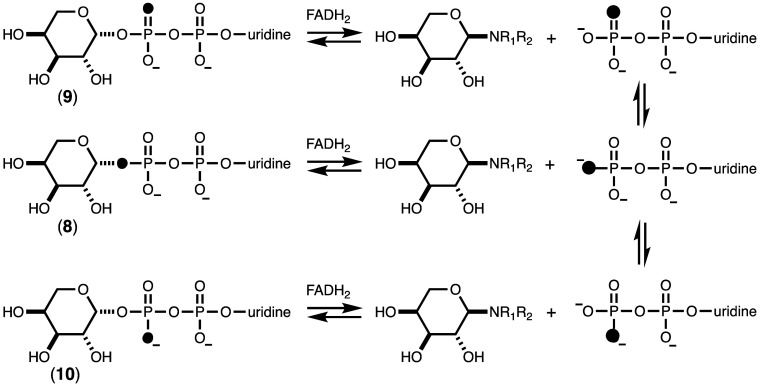
Proposed mechanism for the PIX of ^18^O-labeled UDP-β-l-arabinopyranoside (**8**) catalyzed by UDP-β-l-arabinopyranoside mutase. In the first step, the bound flavin attacks C1 of **8** to reversibly form a flavin/sugar intermediate and UDP. Rotation of β-phosphoryl group of UDP will positionally scramble the ^18^O-label. Upon reformation of UDP-β-l-arabinopyranoside the ^18^O-label will be statistically scrambled among the three possible positions (**8**, **9**, and **10**).

The proposed reaction mechanisms for FADH_2_-dependent pyranose/furanose mutases have postulated the existence of a covalent adduct between N5 of the reduced flavin and C1 of the substrate that is formed via the cleavage of UDP as illustrated in Figure 6.^15,17–19^ The reversible formation of the N5–C1 adduct and UDP can be addressed using an 18-oxygen labeled substrate by monitoring a positional isotopic exchange (PIX) reaction as illustrated in Figure 7.^15,18^ Incubation of the enzyme in the presence of labeled UDP-d-arabinopyranoside (**8**) will form a mixture of UDP and the adduct with FADH_2_. If the β-phosphoryl group of the oxygen-18 labeled UDP is free to rotate, after reformation of the UDP-d-arabinopyranoside, the oxygen-18 label will be positionally scrambled among the two nonbridging positions (structures **9** and **10**) and bridging position (structure **8**). The formation of products **9** and **10** can be monitored by ^31^P NMR spectroscopy since there is a measurable chemical shift difference between compounds **9**/**10** and **8** of approximately 0.02 ppm.^20^

**Figure 8:**
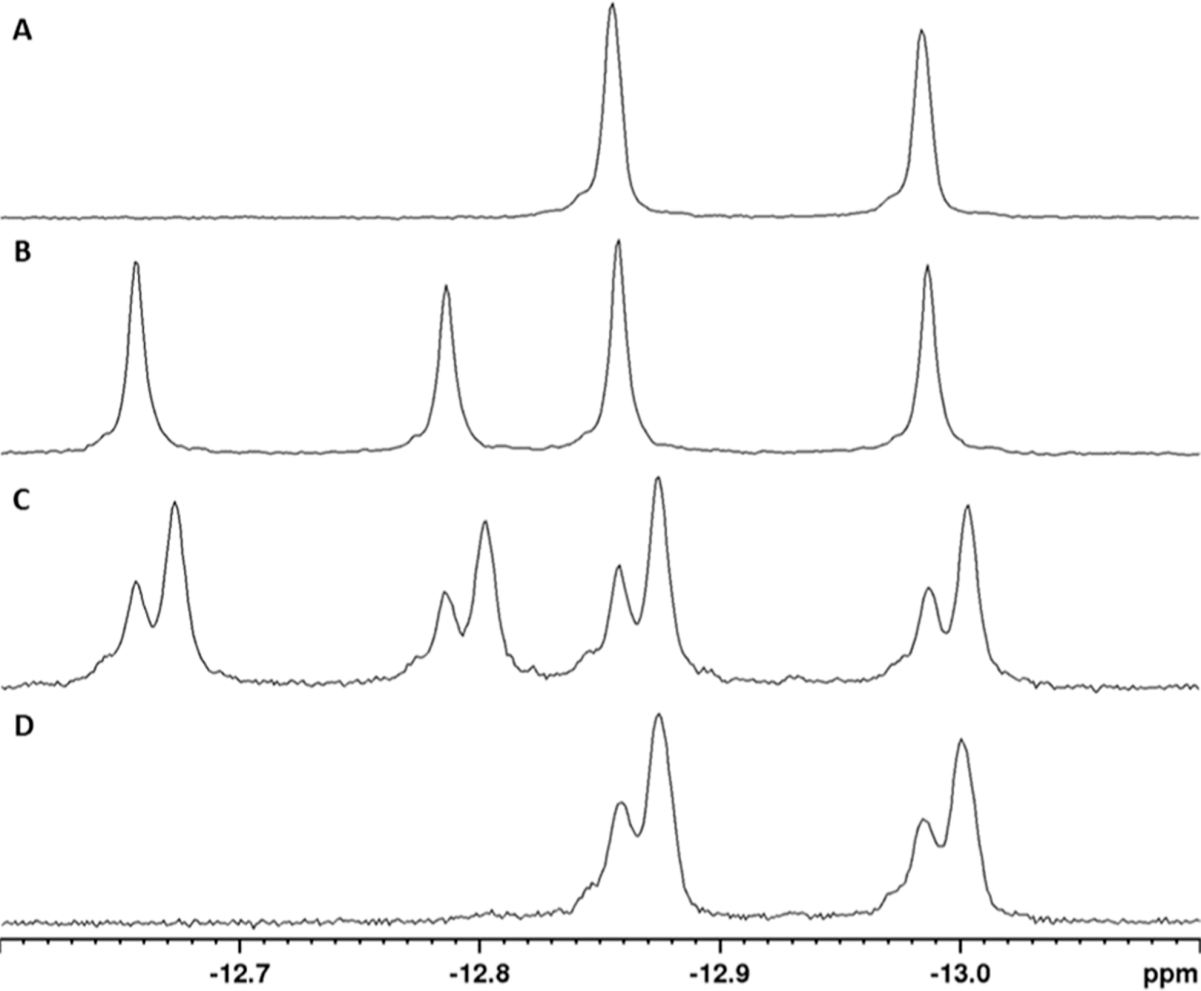
^31^P NMR spectra of the β-phosphoryl groups of UDP-d-xylose and UDP-l-arabinose. (A) β-Phosphoryl group of 18-oxygen labeled UDP-d-xylose. (B) Mixture of oxygen-18 labeled UDP-d-xylose and UDP-l-arabinose (**8**). (C) Equilibrium mixture of oxygen-18 labeled UDP-d-xylose and UDP-l-arabinose (**8**, **9**, and **10**) after the addition of UDP-l-arabinopyranoside mutase. (D) Equilibrium mixture of oxygen-18 labeled UDP-d-xylose after the addition of UDP-l-arabinopyranoside mutase.

Oxygen-18 labeled UDP-d-xylose was synthesized enzymatically according to the procedure outlined in Figure S3. The ESI-mass spectrum of the isolated product has an *m*/*z* value of 537.04 for the M-1 anion. Based on the relative intensity for the peak at an *m*/*z* of 535.04 for the unlabeled UDP-d-xylose, the extent of incorporation of 18-oxygen is 91%. The ^31^P NMR spectrum of the isolated material is presented in Figure 8a showing only the doublet for the β-phosphoryl group. The 18-oxygen labeled UDP-d-xylose was further incubated with the 5.0 nM UDP-d-xylose 4-epimerase to provide an equilibrium mixture of UDP-d-xylose and UDP-l-arabinose. The ^31^P NMR spectrum for the β-phosphoryl group is shown in Figure 8b. This material, after the removal of the UDP-d-xylose 4-epimerase, was added to 1.25 μM UDP-l-arabinopyranoside mutase, and the ^31^P NMR spectrum was obtained after 3 h. The spectrum clearly shows the equilibration of **8**, **9**, and **10**, where the most upfield half of the doublet accounts for approximately 2/3 of the signal intensity with a separation of approximately 0.016 ppm. This result clearly demonstrates that during the UDP-d-arabinopyranoside mutase catalyzed reaction the anomeric carbon–oxygen bond is broken and subsequently reformed. No position isotope exchange was observed in the absence of added dithionate, demonstrating that the flavin must be reduced prior to carbon–oxygen bond breaking. It was also interesting to note that a positional isotopic exchange was also detected within the UDP-d-xylose (Figure 8d). This may have resulted from the incomplete removal of the UDP-d-xylose 4-epimerase, but when we added the UDP-l-arabinopyranoside mutase to the ^18^O-labeled UDP-d-xylose, a PIX reaction was also observed, indicating that the C–O bond cleavage can occur within this substrate. However, we doubt that it would be possible to form the corresponding UDP-d-xylofuranoside product because of the incorrect stereochemistry at C4.

### Biosynthesis of UDP-β-l-Arabinofuranose in Other Serotypes of *C. jejuni*

Homologues of the four enzymes characterized here for the biosynthesis of UDP-β-l-arabinofuranoside (**7**) from the HS:15 serotype of *C. jejuni* can also be found within the CPS gene clusters for the HS:6/7, HS:12. HS:21, HS:29, HS:32/58, HS:33/35, HS:38, HS:40, HS:41, HS:42, HS:55, HS:57, and HS:60 serotypes. The sequence identities of the four proteins from each serotype vary from one another between 81 and 100%. As noted earlier, we identified a 147-base extension at the 5′-end of the gene for the mutase from the HS:15 serotype (UniProt id: F2X7B0) that appeared to interfere with heterologous gene expression in *E. coli*. The same extension was found in the gene from the corresponding mutase from the HS:42 serotype (UnitProt id: F2X7E9). Since this extension does not appear in any of the other mutases from the other serotypes containing the gene cluster for UDP-l-arabinofuranoside production, we have assumed that the proposed start sites for the mutase genes from the HS:15 and HS:42 serotypes have been misannotated. It is also curious to note that the gene cluster for the HS:6/7 serotype has the four genes required for the biosynthesis of UDP-l-arabinofuranoside (**7**), but this sugar is not found within the chemically determined CPS from this strain of *C. jejuni*.^21^ We are uncertain of why this is the case.

## Conclusions

We have identified the four genes required for the biosynthesis of UDP-β-l-arabinofuranoside in the HS:15 serotype of *C. jejuni*. The reaction starts with the NAD^+^-dependent oxidation of UDP-α-d-glucose (**3**) to UDP-α-d-glucuronate (**4**). In the next step, the UDP-α-d-glucuronate is decarboxylated to form UDP-α-d-xylose (**5**) and then this product is isomerized at C4 to form UDP-β-l-arabinose (**6**). In the final step, a FADH_2_-dependent pyranose/furanose mutase converts UDP-β-l-arabinose (**6**) into UDP-β-l-arabinofuranoside. (**7**). The pyranose/furanose mutase was further interrogated by measurement of a PIX reaction using [^18^O]-labeled UDP-β-l-arabinose showing that the mutase catalyzes the transient cleavage of UDP within the substrate prior to the pyranose/furanose transformation. We have identified similar genes in 14 other serotypes of *C. jejuni*.

## Supporting Information Available

Amino acid sequences of the proteins purified for this investigation, synthesis of [^18^O]-UDP-l-arabinopyranoside, chemical synthesis of UDP-β-l-arabinofuranoside, and ^1^H NMR spectra of UDP-α-d-glucuronate (4) and UDP-α-d-xylose (5) + UDP-α-d-xylose/UDP-β-l-arabinopyranose mixture with and without deuterium incorporation (PDF)

## Accession Codes

HS:15.19 (Uniprot F2X7B3); HS:15.18 (Uniprot F2X7B2); HS:15.17 (Uniprot F2X7B1); HS:15.16 (Uniprot F2X7B0); *L. major* UDP-α-d-glucose pyrophosphorylase (Accession EU249268.1).
