# Supplementary material for: Biosynthesis of UDP-β-l-Arabinofuranoside for the Capsular Polysaccharides of Campylobacter jejuni
Source: Biochemistry. 2023 Sep 22;62(20):3012–9. doi: 10.1021/acs.biochem.3c00298 (PMC10615251; doi:10.1021/acs.biochem.3c00298)
Supplement: Supplementary file 1 — bi3c00298_si_001.pdf [file bi3c00298_si_001.pdf]

Supplementary Information

Biosynthesis of UDP-β-L-Arabinofuranoside for the Capsular Polysaccharides of *Campylobacter jejuni*

Max Errickson Simons[ψ], Tamari Narindoshvili[φ] and Frank M. Raushel[ψ, φ, *]

[ψ]Department of Biochemistry & Biophysics, Texas A&M University, College Station, TX 77843, USA

[φ]Department of Chemistry, Texas A&M University, College Station, TX 77843, USA

*Contact Information

e-mail: raushel@tamu.edu

phone 1-979-845-3373

HS:15.19 (UniProt id: F2X7B3)

MGSSHHHHHHSSGLVPRGSHMMRVILITGGSGFLGSNLCKRLLSEGNKIICVDNNYTGRIENIKELLENENF
TFIEHDICEPLKITQKLDQIYNFACPASPPAYQGNHAIKTIKTSVYGAINMLELAKEYNATILQASTSEVYGDP
LVHPQNEEYRGNVNPIGIRACYDEGKRCAESLFFDYHRHEGVDIKIIRIFNTYGENMDPNDGRVVSNFICQAL
SGKDITIYGDGSQTRSFCYVDDLIDIIIKVMNSSKDFQGPINTGNPGEFTIKELAQKVIEKTGSKSKIIYKDLPL
DDPTQRRPDISLAKAKFNWEPKINLDEGLEKTIKYFKEKITEFKG

HS:15.18 (F2X7B2)

MGSSHHHHHHSSGLVPRGSHMMKIGIIGTGYVGLPTGVGLAELGNNVICIDREKSKIDALNNGNLTIYEDNL
EELFHKNVKEGRLKFTTSMQEGIKDADLVIIAVGTPPHPVTKEADMKYIHAAATELADYLTGYTVIATKSTV
PVGTGDDIESLISKKNPNADFDVLSLPEFLREGFAVYDFFNPDRIIVGTNSQRAKAVIEKLYEPFKGKSKLLFV
NRRSSETIKYASNAFLAIKIHYINEMANFCEKAGADILEVAKGMGLDTRIGDRFLNPGPGYGGSCFPKDTLA
MAFMGKQNDIDLTLINVAIKGNEERKNQMSERILNSIKEIKNPKIAILGLAFKDGTDDCRESPAVDIVFKLLE
QKIQICAYDPKAMDLAKQILGNKIDYANSMYEAIKDADVIVILTEWKEFSSLDLKKACDLVKHKKIIDLRNLI
DKSEAIKLGFEYQGIGR

HS:15.17 (F2X7B1)

MGSSHHHHHHSSGLVPRGSHMMKNILVVGGAGYIGSHTLKHLLDNDYNCIVMDNLIYGHKQAIDKRAKFIH
ADLLDTFSLASVFKKEKIDALVHFAAFAYVGESVVNPAKYYQNNIVGTINLLNAMLENNVKDIVFSSTCATY
GEPQYTPIDEKHPQNPINAYGRTKLMIEQVFADYEKAYGLRHISLRYFNAAGASKDGLIGESHEPETHLIPLV
LKAIKGEIPAINIFGNDYDTEDGTCIRDYIHVEDLALAHRLALENLHKFSGCINLGTGIGTSVKEIISAAEIVSG
KKCPINYAPRRDGDPARLYADNKKAKEILSWEAKYTDIKDIIKSAWDWENNRKY

HS:15.16 (F2X7B0)

MMKKVKNLIVGCGLSGAILAERLASKGQEVLIIDKREHIGGNIYDYKDQESNITVHKYGPHVFHTSIKEVWEY
LSRFTKWHYFMYRVKAFIDGKEVNIPFNLDTLHKVFPEYLAKELEQKLISKFAFNSKIPILELKNANDKDLE
FLAEYIYKKVFLGYTVKQWGVKPEELDFSVSARVPVYISRDDRYFIDTYQAIPKDGYTKMIENIINHPLIKVQL
NTDFKDIKKDIEYERLFYTGAIDEFFDYKFGRLPYRSLNIVFETFDKEYMQSGPQINYPENYDFTRSVEYKYY
LDEKSTKTILSYEFPCEYEEGKNERYYPIPNDENQKLYEKYLKESAKLRNVVHFLGRLGDYKYYDMDKAVEG
VLKFIKELLQLEHHHHHH

Figure S1: Protein sequences of enzymes produced for this investigation. The polyhistidine purification tags are shown in yellow .

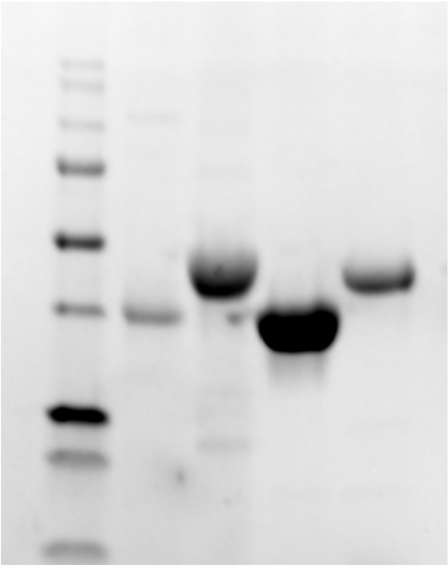

Figure S2: SDS-PAGE assessment of the enzymes purified for this investigation**.** Lane 1: SDS standards protein ladder; Lane 2: UDP-α-D-glucuronate 6-decarboxylase, HS:15.19 (38 kDa); Lane 3: UDP-α-D-glucose 6-dehydrogenase, HS:15.18 (50 kDa); Lane 4: UDP-α-D-xylose 4-epimerase, HS:15.17 (38 kDa); Lane 5: UDP-β-L-arabinopyranose mutase, HS:15.16 (46 kDa).

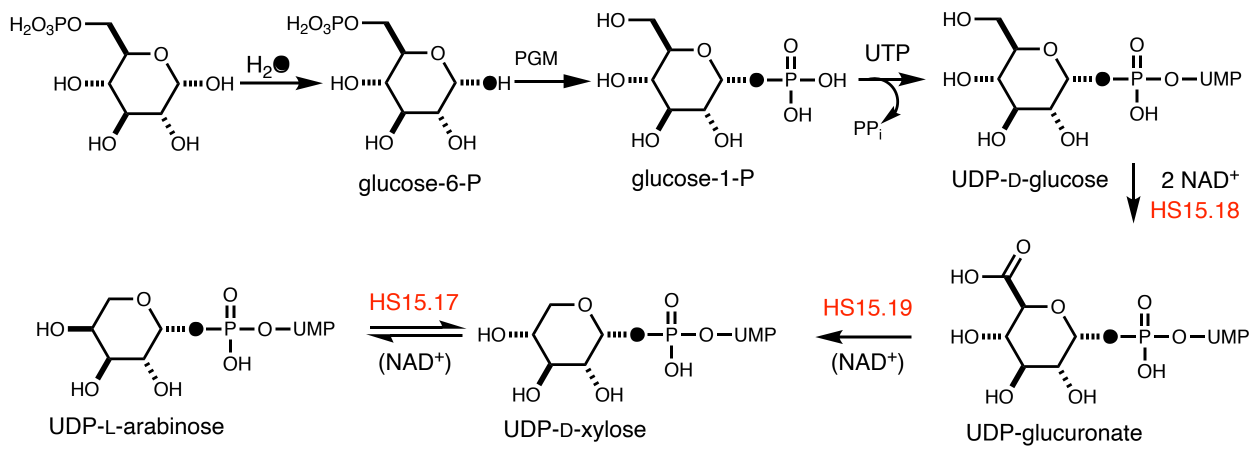

Figure S3: Chemoenzymatic synthesis of oxygen-18 labeled UDP-β-L-arabinose. Additional details are found in the Materials and Methods section of the paper.

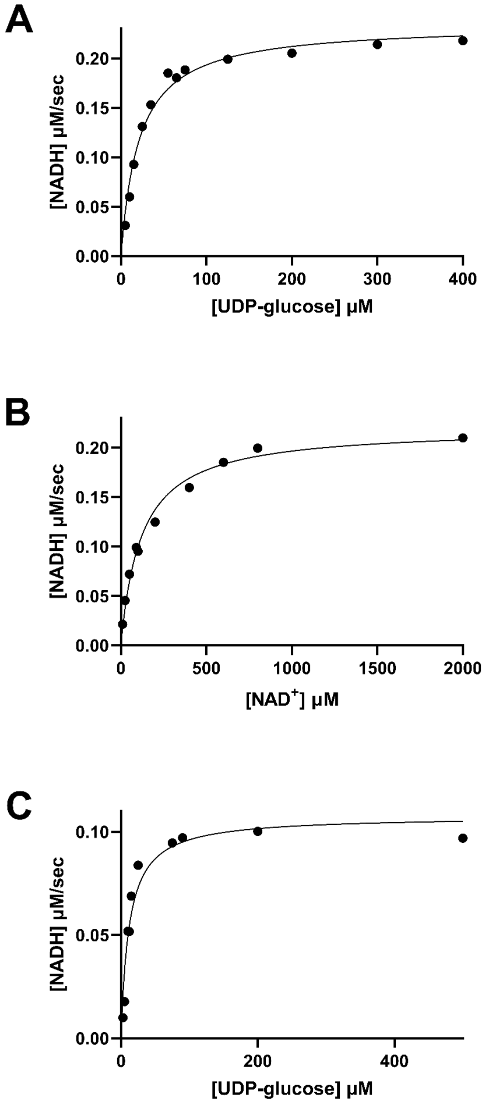

Figure S4: Michaelis-Menten plots for the reaction catalyzed by UDP-α-D-glucose 6-dehydrogenase (HS:15.18). (A) Variation of the concentration of UDP-glucose at a fixed concentration of 1.0 mM NAD^+ at pH 8.7. (B) Variation of the concentration of NAD+ at a fixed concentration of UDP-glucose of 1.0 mM at pH 8.7. (C) Variation of the concentration of UDP-glucose at a fixed concentration of NAD+ of 1.0 mM at pH 8.0. All reactions contained 1.0 mM DTT.

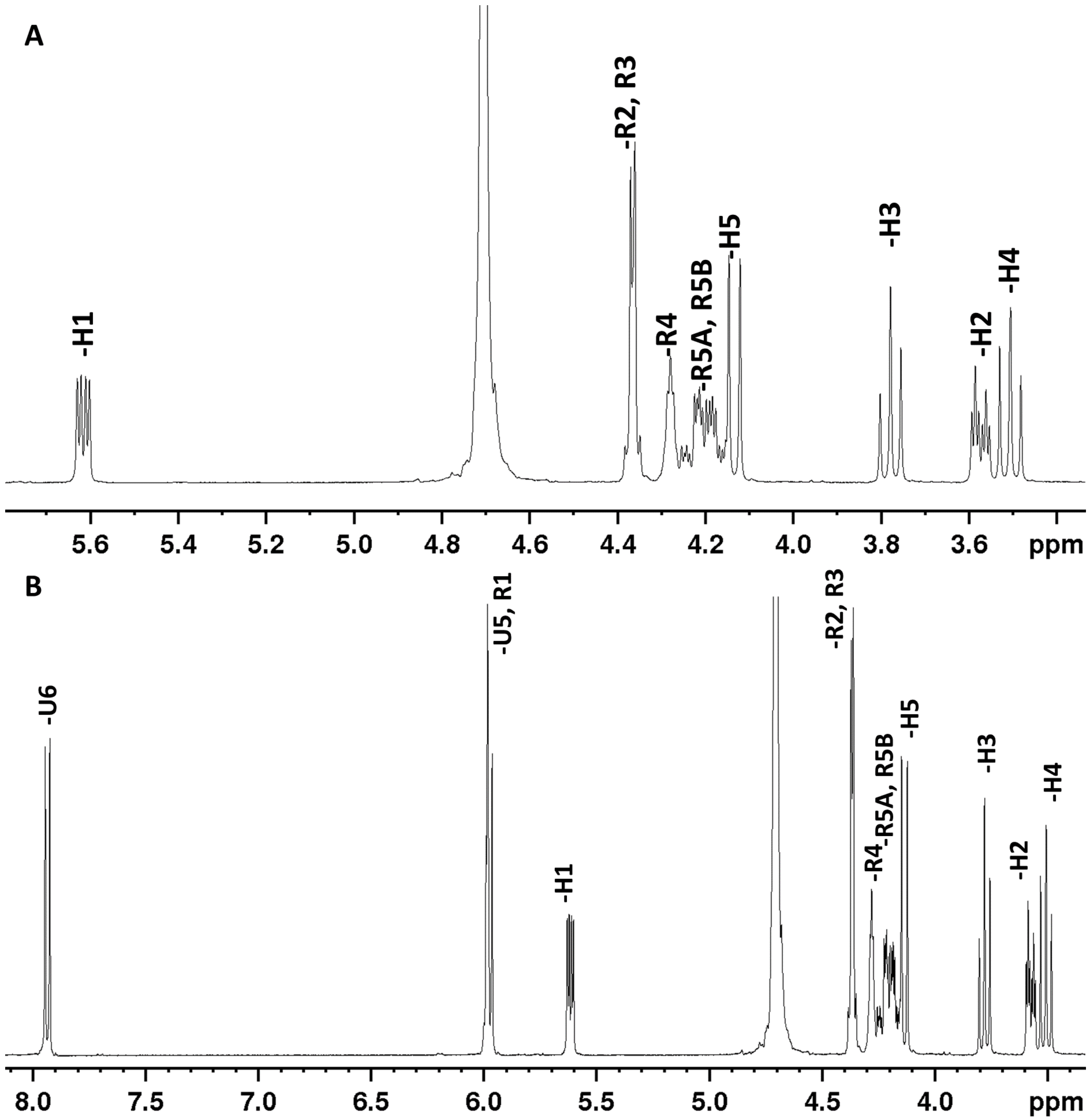

Figure S5: ^{1}H NMR spectrum of UDP-α-D-glucuronate (**4**). The resonances labeled as H1 through H5 are the hydrogens for the glucuronate moiety of **4** while those resonances labeled as R2 through R5A/B are for the ribose moiety. The two hydrogens for the uridine moiety are found at 5.9 and 7.9 ppm and labelled as U5 and U6, respectively.

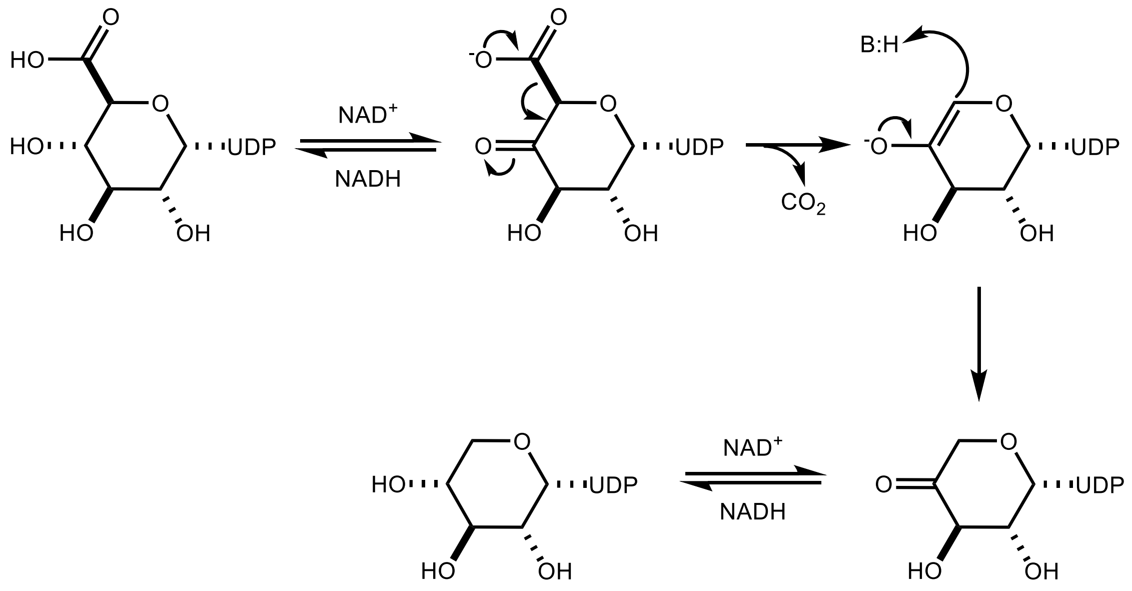

Figure S6: Proposed mechanism for decarboxylation of UDP-α-D-glucuronate (**4**) to UDP-α-D-xylose (**5**). In the proposed mechanism the NAD+ is used to oxidize C4 to form a β-keto acid intermediate. After decarboxylation the keto-group is reduced by the newly formed NADH.[1]

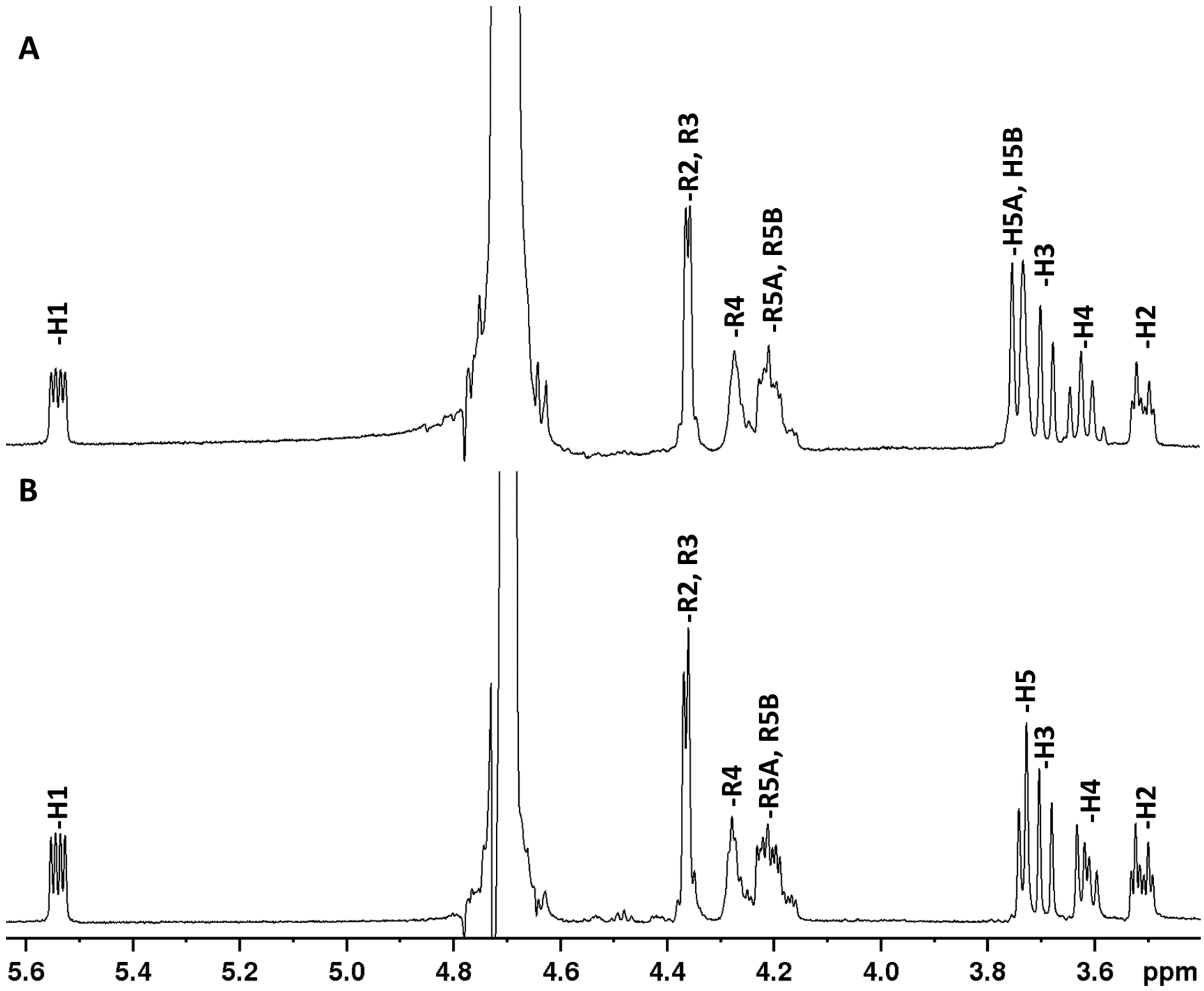

Figure S7: ^{1}H NMR spectra of UDP-α-D-xylose (**5**). (A) UDP-α-D-xylose formed in H_2O by the catalytic activity of UDP-α-D-glucuronate 6-decarboxylase using UDP-α-D-glucuronate (**4**) as the substrate. (B) UDP-α-D-xylose formed in D_2O using UDP-α-D-glucuronate (**4**) as the substrate. The resonances labeled as H1 through H5 are the hydrogens for the xylose moiety of **5** while those resonances labeled as R2 through R5A/B are for the ribose moiety. The two hydrogens for the uridine moiety are found at 5.9 and 7.9 ppm are not shown in this spectrum.

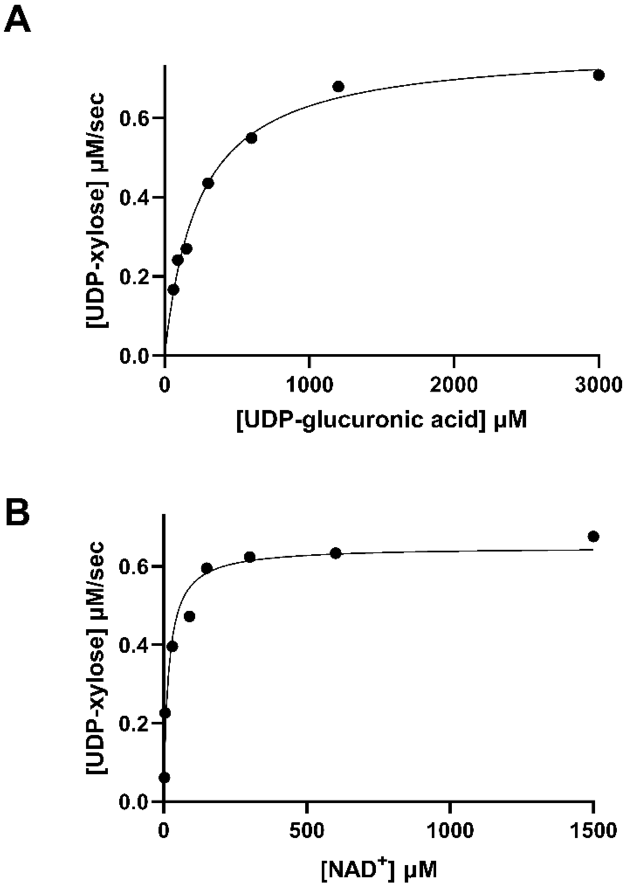

Figure S8: Michaelis-Menten curves for variation of substrate concentration for the reaction catalyzed by UDP-α-D-glucuronate 6-decarboxylase (HS:15.19). (A) Variation of UDP-D-glucuronate at a fixed concentration of 0.3 mM NAD^+ at pH 6.5. (B) Variation of the concentration of NAD^+ at a fixed concentration of 2.5 mM UDP-D-glucuronate at pH 6.5.

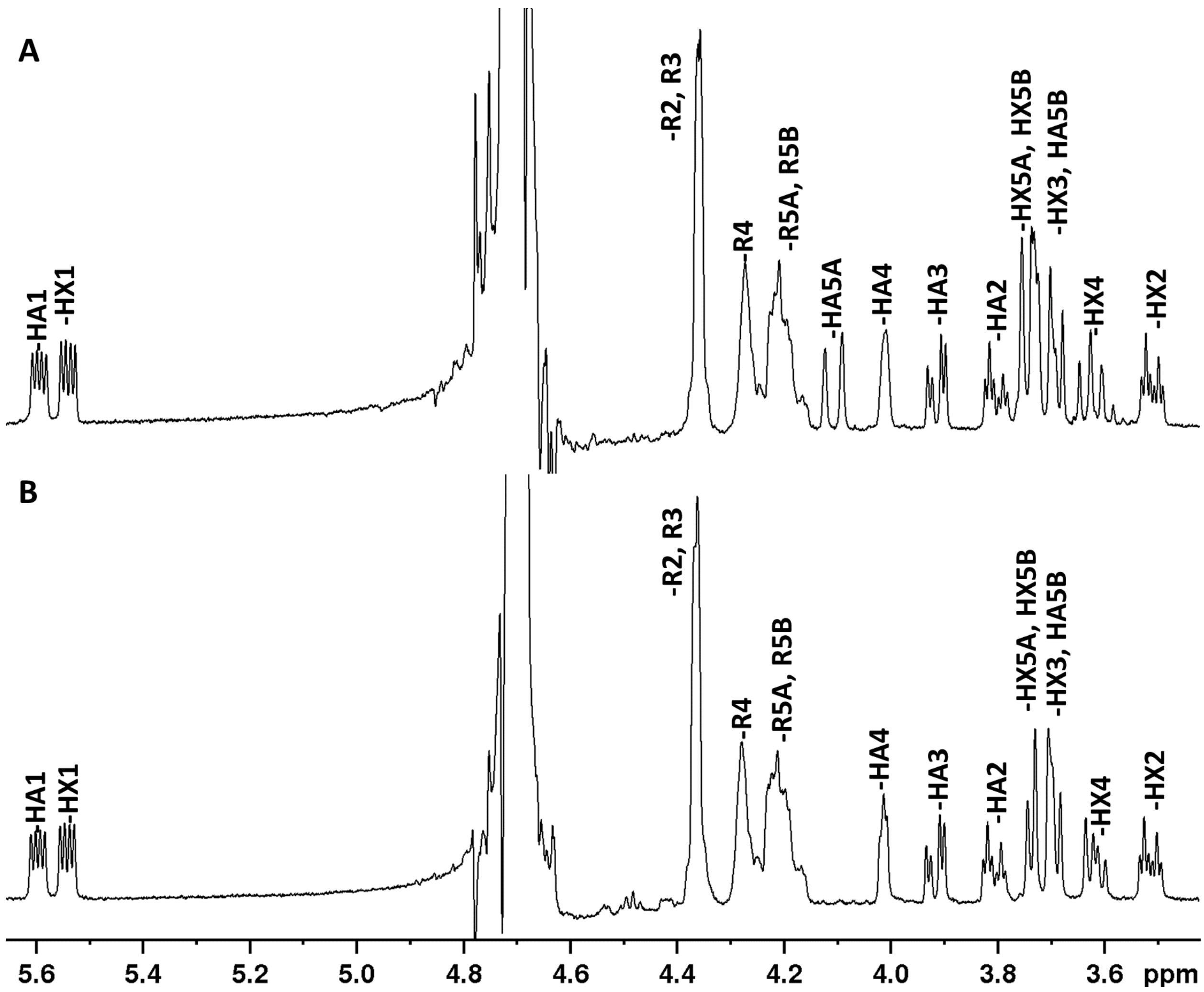

Figure S9: ^{1}H NMR spectra of an equilibrium mixture of UDP-α-D-xylose (**5**) and UDP-β-L-arabinose (**6**) formed in H_2O and D_2O. (A) UDP-β-L-arabinose formed in H_2O by the catalytic activity of UDP-α-D-xylose 4-epimerase using UDP-α-D-xylose (**5**) as the initial substrate. (B) Formation of the product in D_2O. The resonances labeled as HA1 through HA5 are the hydrogens for the L-arabinose moiety of **6** while those resonances labeled as R2 through R5A/B are for the ribose moiety. The resonance labeled as HX1 through HX5 are the hydrogens for the D-xylose moiety. The two hydrogens for the uridine moiety are found at 5.9 and 7.9 ppm and are not shown in this spectrum.

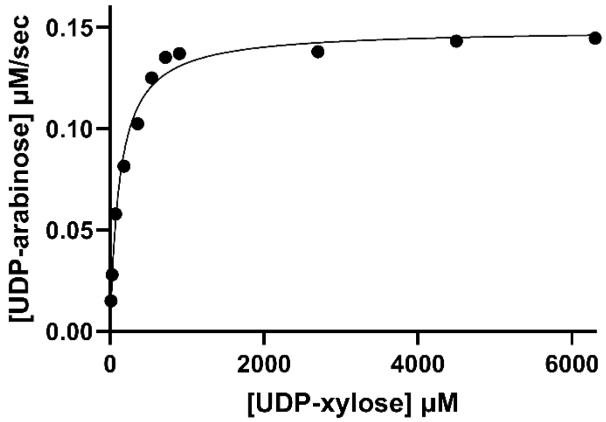

Figure S10: Variation of the UDP-xylose concentration for the reaction catalyzed by UDP-α-D-xylose 4-epimerase (HS:15.17) at pH 8.0.

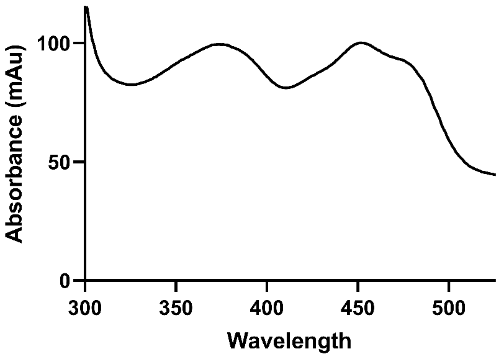

Figure S11: Visible spectrum of the isolated UDP-β-L-arabinopyranoside mutase.

Synthesis of UDP-β-L-Arabinofuranoside (7).

The synthesis of UDP-β-L-arabinofuranoside (**7**) was achieved based on literature procedures.[1-7] The overall synthesis of this compound consists of two parts: preparation of β-L-arabinofuranoside-1-phosphate (**S7**) as illustrated in **Scheme S1**, and preparation of UDP-β-L-arabinofuranoside (**7**) as illustrated in **Scheme S2**.

Synthesis of β-L-Arabinofuranoside 1-Phosphate.

β-L-Arabinofuranoside-1-phosphate (**S7**) was previously reported to be synthesized from L-arabinose (**S1**) in several steps.[1, 6, 7] L-Arabinose (**S1**) was converted into a mixture of α- and β-isomers (α:β, 1:5.6) of methyl-L-arabinofuranoside (**S2**) according to a procedure previously described for the preparation of methyl-D-arabinofuranoside.[2] An anomeric mixture of 1,2,3,5-tetra-*O*-acetyl-L-arabinofuranoside (**S3**) (α/β,1:4.5) was prepared from **S2** using direct acetylation, followed by acetolysis of the methyl group.[3-5] Treatment of **S3** with 33% HBr/AcOH and Ac_2O in dichloromethane formed **S4**.[6] Subsequent phosphorylation of **S4** was conducted in anhydrous benzene with one equivalent of triethylammonium dibenzyl phosphate.[1,6] A fully protected L-arabinofuranoside 1-phosphate, as the β-isomer (**S5**) was isolated by silica gel chromatography in 10% yield.[1] From **S5**, the triacetyl protected β-L-arabinofuranoside 1-phosphate was obtained as the mono triethylammonium salt (**S6**) by catalytic hydrogenation, applying 10% Pd/C catalyst in mixture of ethyl acetate/triethylamine (18:1). Deacetylation of **S6** was conducted in a mixture of MeOH/H_2O/TEA (5:2:1) to obtain the fully deprotected β-L-arabinofuranoside 1-phosphate bis-triethylammonium salt (**S7**).

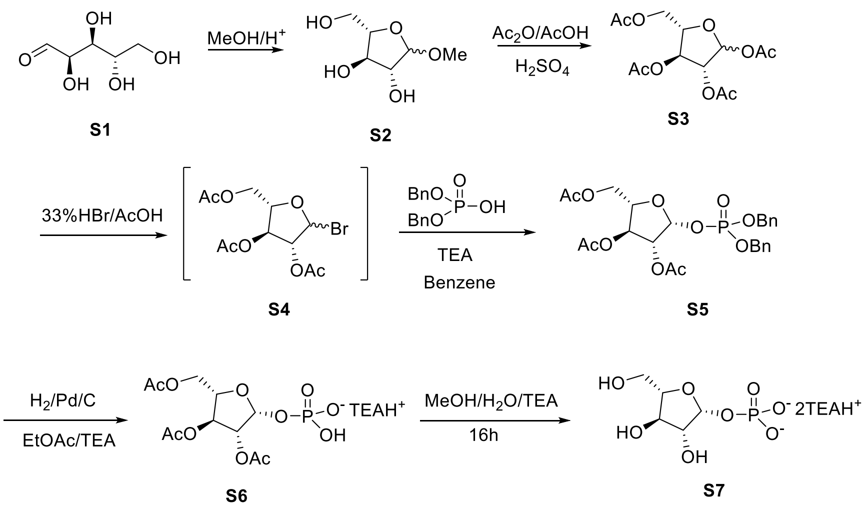

Scheme S1: Chemical synthesis of L-arabinose-1-phosphate

Synthesis of UDP-β -L-arabinofuranoside

UDP-β-L-arabinofuranose (**7**) was synthesized by coupling 1 equivalent of β-L-arabinofuranosyl 1-phosphate (**S7**) with 2 equivalents of uridine 5'-phosphoromorpholidate dicyclohexylcarboxamidine salt (**S8**) using 1.25 equivalents of 4,5-dicyanoimidazole (DCI) as illustrate in **Scheme S2**.[7] Synthetic procedure and purification details are described below.

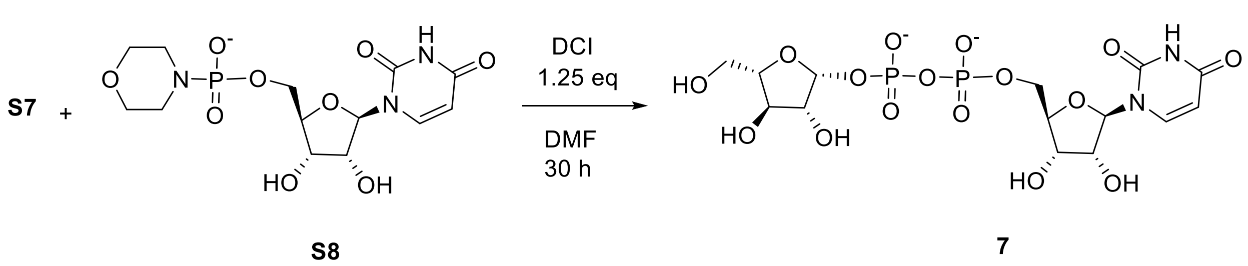

Scheme S2: Chemical synthesis of UDP-β-L-arabinofuranoside.

Synthetic procedures:

Methyl-L-arabinofuranoside (*S*2).

L-Arabinose (1.5 g, 10 mmol) was dissolved in MeOH (35 mL) and then acetyl chloride (0.21 mL) was added and the reaction stirred overnight. The reaction mixture was quenched by adjusting the pH to 6.5 (by carefully adding of solid $NaHCO_3$) and concentrated under reduced pressure. The residue was co-evaporated with toluene before being purified by flash chromatography (EtOAc/MeOH; 20:1) yielding an anomeric mixture (α:β; 1:5.6) of methyl-L-arabinofuranoside (0.78 g, 47.5 %).

α-Anomer: ^{1}H NMR (400 MHz, MeOH-d$_4$) δ (the signal for the anomeric proton is under the water peak), 3.98 – 3.87 (m, 2H), 3.82 – 3.78 (m, 1H), 3.72-3.68 (m, 1H), 3.59-3.59 (m, 1H), 3.43 (s, 3H) ppm. Shown in **Figure S12**.

^{13}C NMR (125 MHz, MeOH-d$_4$) δ 104.0 (C-1), 84.3, 78.7, 76.8, 65.4, 55.5 ppm. Shown in **Figure S13**.

β-Anomer: ^{1}H NMR (400 MHz, MeOH-d$_4$) δ 4.78 (d, J = 1.4 Hz, 1H), 3.97 – 3.89 (m, 2H), 3.86 – 3.83 (m, 1H), 3.76 (dd, J_1 = 3.2 Hz, J_2 = 11.9 Hz, 1H), 3.65 (dd, J_1 = 5.3 Hz, J_2 = 11.9 Hz, 1H), 3.38 (s, 3H) ppm. Shown in **Figure S12**.

^{13}C NMR (125 MHz, MeOH-d$_4$) δ 110.5, 85.5, 83.2, 78.7, 63.0, 55.2 ppm. Shown in **Figure S13**.

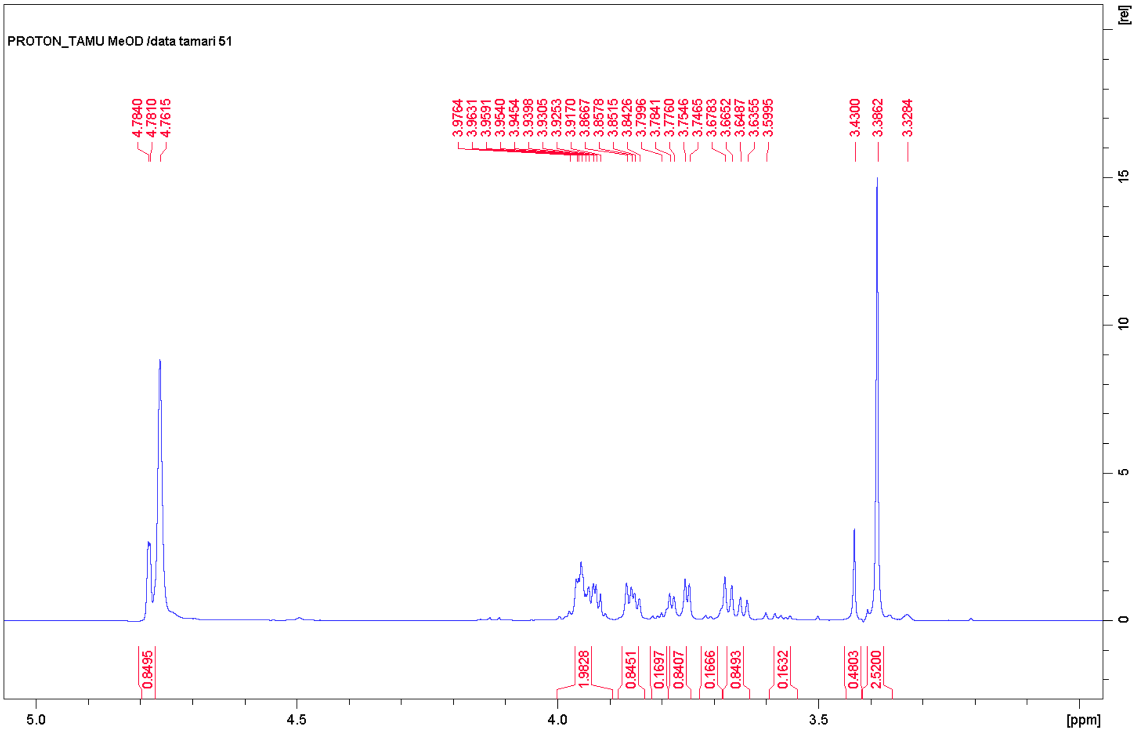

Figure S12: ^{1}H spectrum of the α/β-anomers of **S2** in MeOH-d*4.*

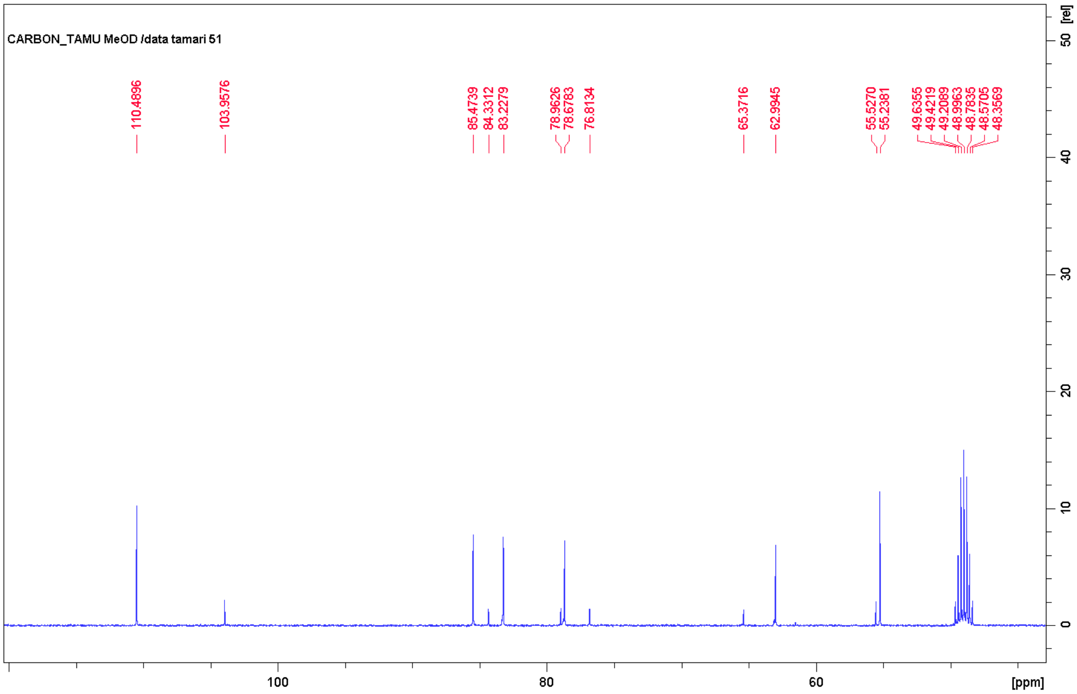

Figure S13: ^{13}C spectrum of the α/β mixture of **S2** in MeOH-d_4.

1,2,3,5-Tetra-*O*-acetyl-β-L-arabinofuranose (*S*3).

A solution of **S2** (2.9 g, 17.7 mmol) in glacial AcOH (7.5 mL) and Ac_2O (10 mL) was cooled (ice bath) and H_2SO_4 (0.3 mL) was added. The reaction was stirred at room temperature for 1 h and then placed in an ice bath and a second portion of H_2SO_4 (0.5 mL) was added. The reaction was stirred for 2 h at room temperature and then quenched by the addition of NaOAc (2.5 g). The reaction mixture was evaporated and the brownish residue was dissolved in dichloromethane (50 mL), washed with water (3 × 50 mL), dried with Na_2SO_4, and the solvent evaporated to dryness. An anomeric mixture of 1,2,3,5-tetra-*O*-acetyl-L-arabinofuranose (α/β,1:4.5) was obtained as a colorless oil (3.8 g, 67%).

α-Anomer: ^{1}H NMR (400 MHz, $CDCl_3$) δ 6.40 (d, *J* = 4.0 Hz, 1H), 5.23 (d, *J* = 1.6 Hz, 1H), 5.39-5.36 (m, 2H), 4.43-4.21 (m, 3H), 2.13, 2.11, 2.10 (3s, 12H) ppm. Shown in **Figure S14**.

^{13}C NMR (125 MHz, $CDCl_3$) δ 170.5, 170.2, 169.6, 169.2, 93.7, 79.6, 75.3, 74.8, 64.5, 21.0, 20.7, 20.4 ppm. Shown in **Figure S15**.

β-Anomer: ^{1}H NMR (400 MHz, $CDCl_3$) δ 6.21(s, 1H), 5.23 (d, *J* = 1.6 Hz, 1H), 5.09-5.06 (m, 1H), 4.44-4.35 (m, 2H), 4.31-4.21(m, 1H), 2.24 (s, 3H), 2.14 (s, 3H), 2.13(s, 3H), 2.12(s, 3H) ppm. Shown in **Figure S14**.

^{13}C NMR (125 MHz, $CDCl_3$) δ 170.5, 169.9, 169.4, 169.2, 99.3, 82.4, 80.6, 76.8, 63.0, 22.1, 21.0, 20.7, 20.6 ppm. Shown in **Figure S15**.

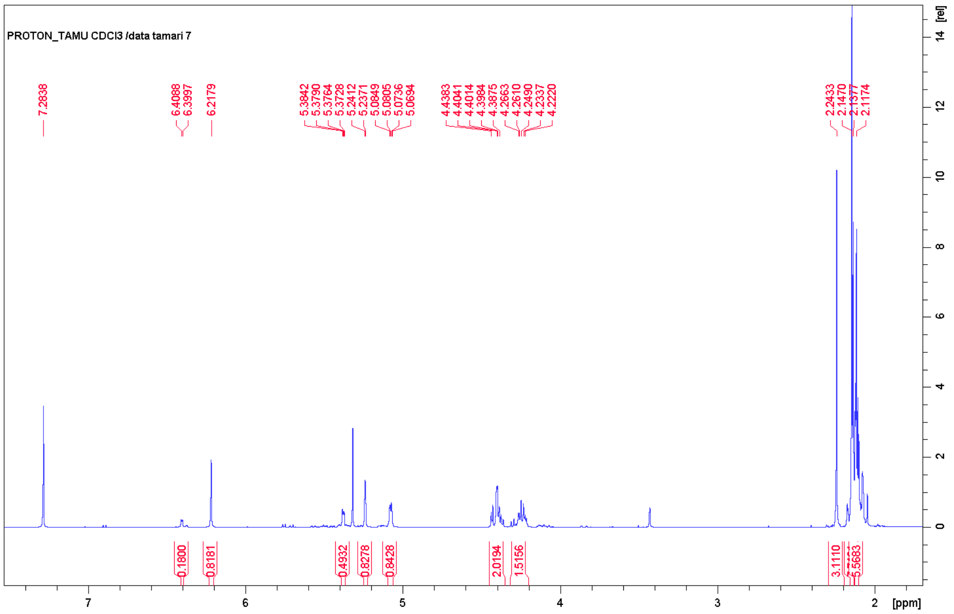

Figure S14: ^{1}H spectrum of an α/β mixture of compound **S3** in $CDCl_3$.

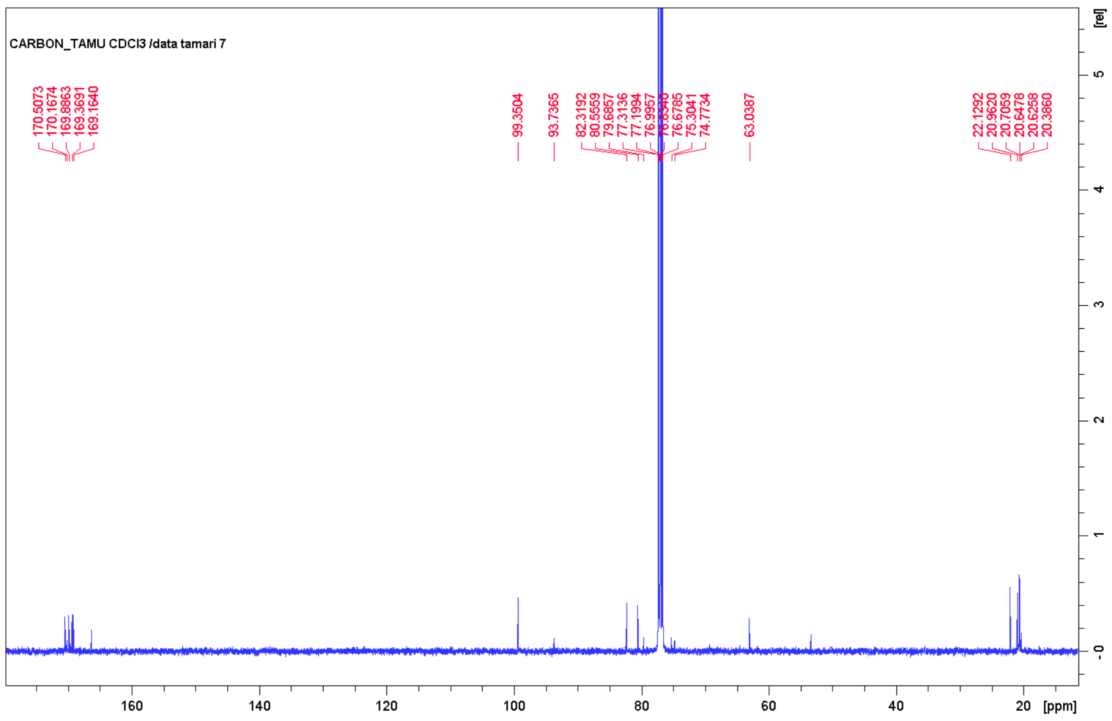

Figure S15: ^{13}C spectrum of an α/β mixture of compound **S3** in $CDCl_3$

Synthesis of Dibenzyl (2,3,5-tri-O-acetyl- β-L-arabinofuranoside) 1-phosphate (S5).

To a solution of 1.0 g (3.1 mmol) of 1,2,3,5-tetra-O-acetyl-D-arabinofuranose **S3** in 5 mL of dry dichloromethane were added 3 mL of 33% hydrogenbromide in acetic acid and 0.2 mL of acetic anhydride; the solution was stirred at room temperature for 1 h. The solvent and the excess of reagent were evaporated *in vacuo* and the last traces removed by co-evaporation with toluene *in vacuo*. The residual bromide was dissolved in 5 mL of dry benzene and to the solution was added a benzene (3 mL) solution of 1.1 equivalent of triethylammonium dibenzyl phosphate (mixture of 1.1 equivalent of dibenzyl phosphate and 1.1 equivalent of triethylamine). After 1 h at room temperature, the crystalline triethylammonium bromide was filtered and washed with dry benzene. The combined filtrates were concentrated under reduced pressure and subjected to silica gel column chromatography (hexanes/ethyl acetate, 5:3) to obtain the desired product **S5,** as a single isomer: dibenzyl (2,3,5-tri-O-acetyl- β-L-arabinofuranoside 1-phosphate (0.17 g, 0.32 mmol) as a colorless oil (10% yield). Due to numerous byproducts and instability, several column purifications were necessary to obtain pure **S5**.

^{1}H NMR (400 MHz, $CDCl_3$) δ 7.33-7.22 (m, 10H), 5.94 (dd, $J_{1,2}$ = 4.5 Hz, $J_{H1,P}$ = 5.4 Hz,1H), 5.36-5.31 (m, 1H), 5.20-5.15 (m, 1H), 5.02-4.94 (m, 4H), 4.36 (dd, J_1 = 3.6, J_2 = 11.4 Hz, 1H), 4.18-4.04(m, 2H) ppm. Shown in **Figure S16**.

^{13}C NMR (125 MHz, $CDCl_3$) δ 170.5, 170.0, 169.9, 135.6, 135.5, 128.6, 127.7, 97.8 (d, J = 5.0 Hz, 1C), 79.9, 75.9 (d, J = 6.9 Hz, 1H), 74.1 (s, 1P), 69.4 (t, J = 5.2Hz, 2C), 64.4, 20.7, 20.6, 20.3. Shown in **Figure S17**.

^{31}P NMR (160 MHz, $CDCl_3$) δ – 2.89 (s) ppm. Shown in **Figure S18**.

(ESI$^-$) m/z [M + H]$^+$ calcd. for $C_{25}H_{30}O_{11}P$: 537.1526, found: 537.1534.

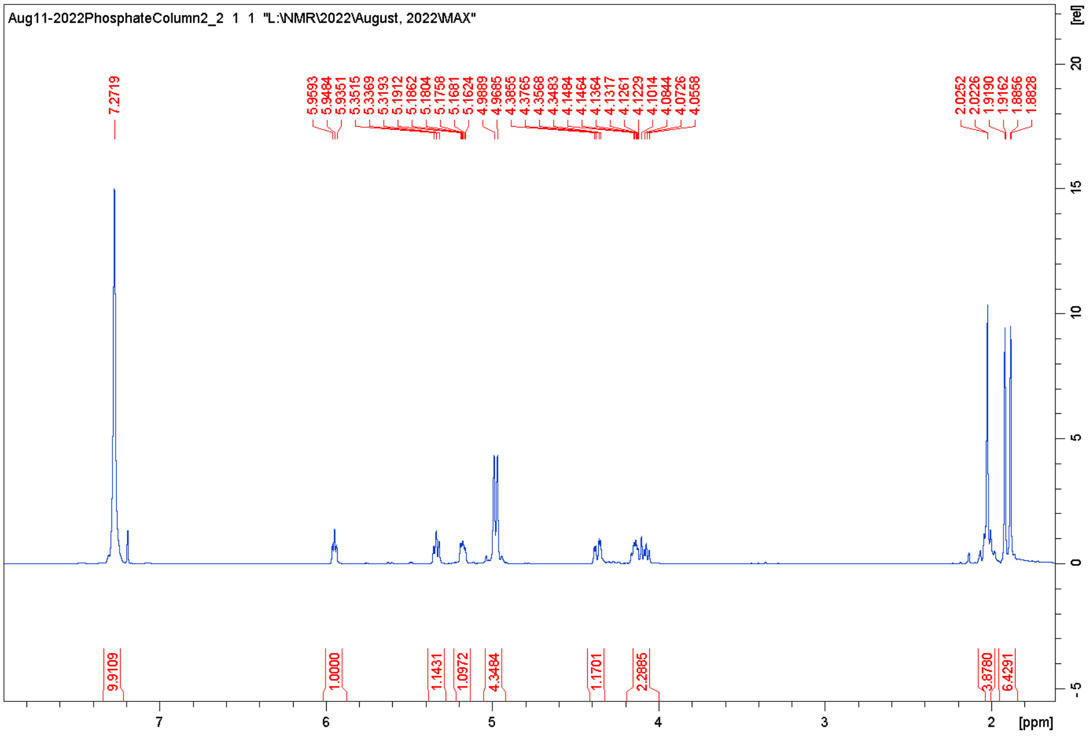

Figure S16: ^1H spectrum of **S5** in $CDCl_3$.

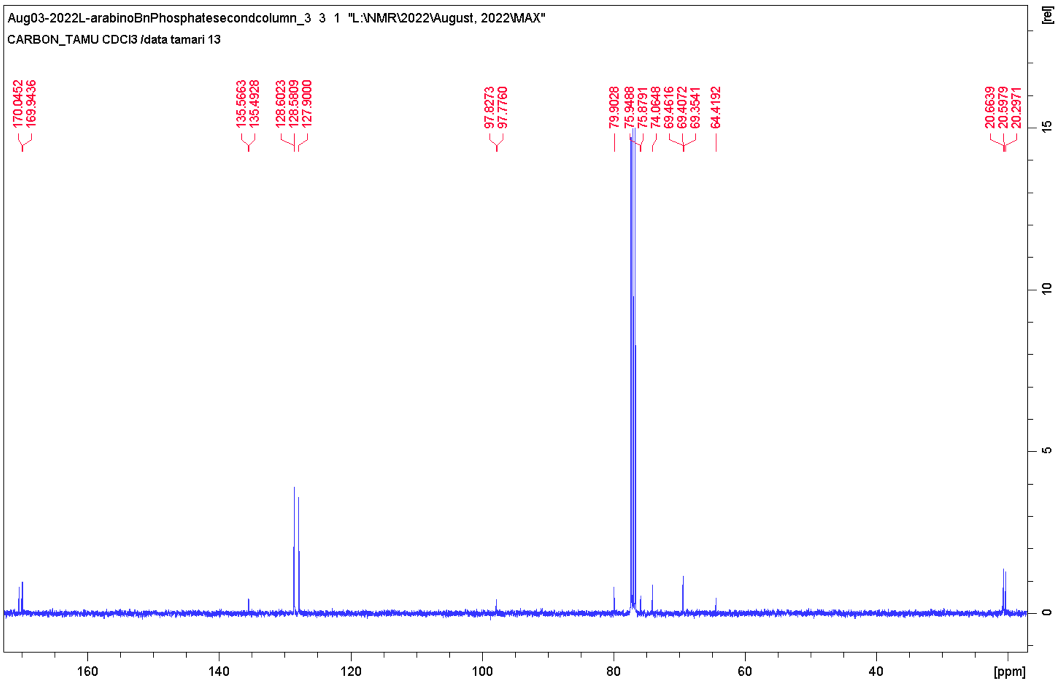

Figure S17: ^{13}C spectrum of **S5** in $CDCl_3$.

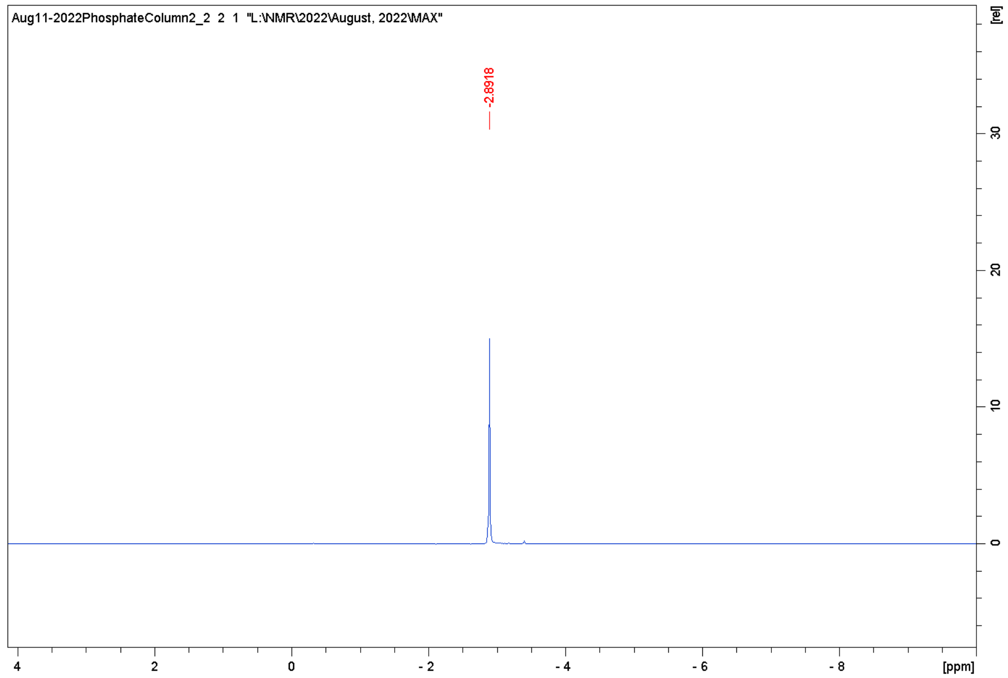

Figure S18: ^{31}P spectrum of **S5** in $CDCl_3$.

2,3,5-tri-O-acetyl- β-L-arabinofuranosyl phosphate monotriethylammonium salt (S6)

To the solution of **S5** (0.17 g, 0.32 mmol) in 18 mL of ethyl acetate and 1 mL of triethylamine, Pd/C (10%) (0.10 g) was added and the reaction mixture was subjected to H_2 (1 atm) for 24 h. After filtering and concentrating, **S6** (0.13 g, 0.28 mmol) was obtained (89% yield).

^{1}H NMR (400 MHz, $CDCl_3$) δ 5.79 (dd, $J_{1,2}$ = 4.2 Hz, $J_{H1,P}$ = 7.0 Hz,1H), 5.39-5.34 (m, 1H), 5.13-5.08 (m, 1H), 4.41 (dd, J_1 = 5.2 Hz, J_2 = 11.4 Hz,1H), 4.21-4.14 (m, 1H), 4.08-4.01 (m, 1H), 2.99 (q, J = 7.2 Hz, 6H), 2.03 (s, 3H), 2.00 (s, 3H), 1.98 (s, 3H), 1.25 (t, J = 7.2 Hz, 9H) ppm.

^{31}P NMR (160 MHz, $CDCl_3$) δ – 0.57 (s) ppm.

β-L-arabinofuranosyl phosphate bistriethylammonium salt (S7)

The solution of **S6** (0.13 g, 0.32 mmol) in 5 mL of methanol, 2 mL of water and 1 mL of triethylamine was kept at room temperature for 16 h. After concentrating, **S7** (0.094 mg, 0.32 mmol) was obtained.

^{1}H NMR (400 MHz, D_2O) δ 5.50 (t, J = 5.5 Hz, 1H), 4.16-4.08 (m, 2H), 3.91-3.84 (m, 1H), 3.78 (dd, J_1 = 2.7 Hz, J_2 = 12.6 Hz,1H), 3.66 (dd, J_1 = 5.6 Hz, J_2 = 12.6 Hz,1H), 3.18 (q, J = 7.2 Hz, 12H), 1.26 (t, J = 7.2 Hz, 18H) ppm.

^{31}P NMR (160 MHz, D_2O) δ + 0.33 (s) ppm.

(ESI$^-$) m/z [M-H]$^-$ calcd. for $C_5H_{10}O_8P$: 229.0113, found: 229.0112.

UDP-β-L-arabinofuranoside bis(triethylammonium) salt (7).

To a solution of uridine 5'-phosphoromorpholidate (95.5 mg, 0.14 mmol, 2 equiv) and β-L-arabinofuranoside 1-phosphate bis-triethylammonium salt (30 mg, 0.07 mmol, 1 equiv) in dry DMF (0.5 mL) was added 4,5-dicyanoimidazole (DCI) (9.8 mg, 0.083 mmol, 1.25 equiv). The reaction was stirred at 23 °C for 30 h. Then, the solution was concentrated *in vacuo*. The residue was extracted with 500 mM TEAB buffer (3 times) (pH = 7.5), and combined solution (1.5 mL) was frozen at -80 °C and. Product **7** was purified using a 5 mL HiTrap™QHP anion exchange column, 5

mL with a linear gradient of 0% to 100%, 500 mM TEAB buffer (pH = 7.5). Fractions with desired product were combined and lyophilized to obtain **7**.

^{1}H NMR (400 MHz, D_2O) δ 7.88 (d, J = 8.1 Hz, 1H), 5.88 (d, J = 8.4 Hz, 2H), 5.56 (dd, J_1 = 3.5 Hz, J_2 = 5.7 Hz,1H), 4.35-4.26 (m, 2H), 4.23–4.14 (m, 3H), 4.08-4.05 (d, m, 2H), 3.87-3.81 (m, 1H), 3.72 (dd, J_1 = 3.0 Hz, J_2 = 12.6 Hz, 1H), 3.62 (dd, J_1 = 6.2 Hz, J_2 = 12.6 Hz, 1H), 3.12 (q, J = 7.2 Hz, 12H), 1.20 (t, J = 7.2 Hz, 18H) ppm. Shown in **Figure S19**.

^{31}P NMR (162 MHz, D_2O) δ −11.3 (d, *J* = 20.0 Hz, 1P), −12.7 (d, *J* = 20.0 Hz, 1P) ppm. Shown in **Figure S20**.

(ESI^-) m/z $[M - H]^-$ calcd. for $C_{14}H_{21}N_2O_{16}P_2$: 535.0366, found: 535.0373.

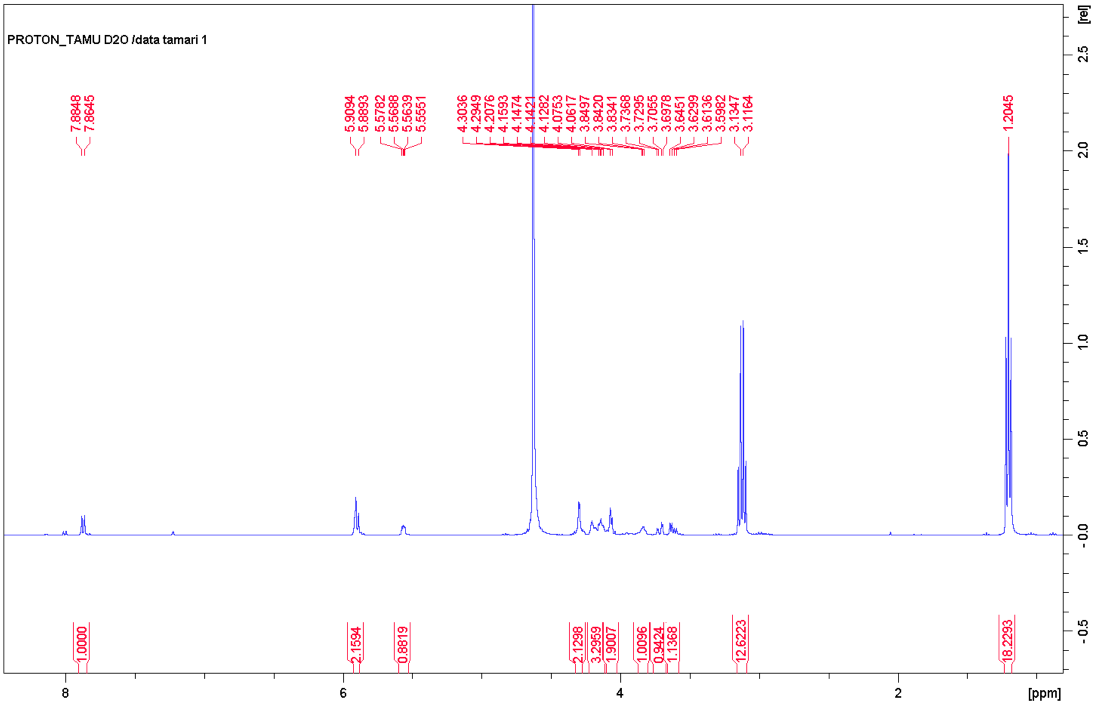

Figure S19: ^{1}H spectrum of **7** in D_2O. The resonance at 1.20 ppm and 3.12 ppm are from triethyl amine.

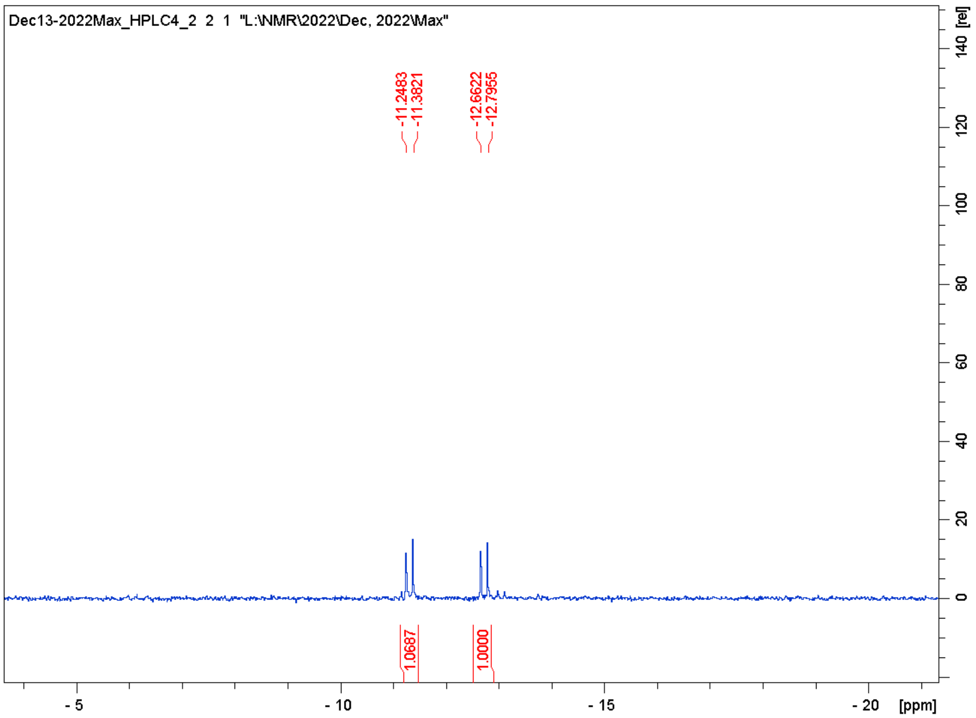

Figure S20: ^{31}P spectrum of **7** in D_2O.

REFERENCES

1. Zhang, Q., Liu, H. (**2001**) Chemical synthesis of UDP-β-l-arabinofuranose and its turnover to UDP-β-l-arabinopyranose by UDP-galactopyranose mutase. *Bioorg. and Med. Chem. Let. 11*, 145-149.
2. van Rijssel, E. R., van Delft, P., Lodder, G., Overkleeft, H. S., van der Marel, G. A., Filippov, D. V., Codee, J. D. (**2014**) Furanosyl Oxocarbenium Ion Stability and Stereoselectivity. *Angew. Chem. Int. Ed. 53*, 10381-10385.
3. Kam, B. L., Barascut, J-L., Imbach, J-L. (**1979**) A general method of synthesis and isolation, and an n.m.r-spectroscopic study, of tetra-O-acetyl-d-aldopentofuranoses. *Carbohydr. Res. 69*, 135-142.
4. Dureau, R., Legentil, L., Daniellou, R., Ferrières, V. (**2012**) Two-Step Synthesis of Per-O-acetylfuranoses: Optimization and Rationalization. *J. Org. Chem. 77*, 1301-1307.
5. Forsman, J. J., Warna, J., Murzin, D. Y., Leino, R. (**2009**) Reaction Kinetics and Mechanism of Sulfuric Acid-Catalyzed Acetolysis of Acylated Methyl L-Ribofuranosides. *Eur. J. Org. Chem. 32*, 5666-5676.
6. Wright, R. S., Khorana, H. G. (**1958**) Phosphorylated Sugars. Syntheses of Arabinofuranose and Arabinopyranose 1-Phosphates. *J. Am. Chem. Soc. 80*, 1994-1998.
7. Chen, W.-J., Han, Sh-B., Xie, Zh-B., Huang, H-Sh., Jiang, D-H., Gong, Sh-Sh., Sun. Q. (**2019**) Efficient Synthesis of UDP-Furanoses via 4,5-Dicyanoimidazole(DCI)-Promoted Coupling of Furanosyl-1-Phosphates with Uridine Phosphoropiperidate. *Molecules, 24,* 655.
